# Synthesis and tyrosinase inhibitory activities of novel isopropylquinazolinones

**DOI:** 10.1186/s13065-023-00978-3

**Published:** 2023-06-23

**Authors:** Arshia Hashemi, Milad Noori, Navid Dastyafteh, Seyed Esmaeil Sadat-Ebrahimi, Negin Fazelzadeh Haghighi, Katayoun Mehrpour, Elahe Sattarinezhad, Fatemeh Jalali Zafrei, Cambyz Irajie, Mohammad Ali Daneshmehr, Majid Heydari, Bagher Larijani, Aida Iraji, Mohammad Mahdavi

**Affiliations:** 1 Department of Medicinal Chemistry, Faculty of Pharmacy and Pharmaceutical Sciences Research Center, Tehran University of Medical Sciences, Tehran, Iran; 2 Endocrinology and Metabolism Research Center, Endocrinology and Metabolism Clinical Sciences Institute, Tehran University of Medical Sciences, Tehran, Iran; 3 Molecular Dermatology Research Center and Department of Dermatology, Shiraz University of Medical Sciences, Shiraz, Iran; 4 Stem Cells Technology Research Center, Shiraz University of Medical Sciences, Shiraz, Iran; 5 Central Research Laboratory, Shiraz University of Medical Sciences, Shiraz, Iran; 6 Department of Pharmacology, School of Medicine, Shiraz University of Medical Sciences, Shiraz, Iran; 7 Cardiovascular Diseases Research Center, Department of Cardiology, Heshmat Hospital, School of Medicine, Guilan University of Medical Sciences, Rasht, Iran; 8 Department of Medical Biotechnology, School of Advanced Medical Sciences and Technologies, Shiraz University of Medical Sciences, Shiraz, Iran; 9 Student Research Committee, Shiraz University of Medical Sciences, Shiraz, Iran

**Keywords:** Isopropylquinazolinone, Synthesise, Tyrosinase, Enzyme inhibition, Kinetic evaluation, In silico studies

## Abstract

To find new anti-browning and whitening agents in this study, new series of isopropylquinazolinone derivatives were designed and synthesized. All derivatives were evaluated as possible tyrosinase inhibitors and compound **9q** bearing 4-fluorobenzyl moieties at the R position exhibited the best potencies with an IC_50_ value of 34.67 ± 3.68 µM. The kinetic evaluations of **9q** as the most potent derivatives recorded mix-type inhibition. Compounds **9o** and **9q** also exhibited potent antioxidant capacity with IC_50_ values of 38.81 and 40.73 µM, respectively confirming their antioxidant potential. Molecular docking studies of **9q** as the most potent derivative were exacuated and it was shown that quinazolinone and acetamide moieties of compound **9q** participated in interaction with critical His residues of the binding site. The obtained results demonstrated that the **9q** can be considered a suitable pharmacophore to develop potent tyrosinase inhibitors.

**Supplementary Information:**

The online version contains supplementary material available at 10.1186/s13065-023-00978-3.

## Introduction

Melanin pigment is produced by the complex melanogenesis process in melanocytes and plays a significant role in determining the color of human skin, eyes, and hair [1]. Melanin also protects humans against the harmful effects of ultraviolet (UV) [2]. On the other hand, excess secretion of melanin from melanocytes leads to dermatologic disorders such as melasma, freckles, post-inflammatory melanoderma vitiligo, and cancer. Also, there are correlations between melanogenesis disorders and neurodegenerative diseases, including Parkinson’s, Alzheimer’s, and Huntington’s diseases [3, 4].

Therefore, more attention has been paid to develop novel agents and reduce the side effects of hyperpigmentation. Tyrosinase (EC.1.14.18.1) is known as the main rate-limiting enzyme that regulates the biosynthesis of melanin production by the conversion of L-tyrosine to L-dopa and then dopaquinone via monophenolase and diphenolase activities [5, 6]. In this context, tyrosinase inhibitors represent key inhibitory agents to overcome the excess production of melanin. A wide variety of standard tyrosinase inhibitors such as kojic acid, tropolone, and hydroquinone are clinically effective. Most of these inhibitors have associated toxic effects and low efficacy problems. Thus, the production of efficient tyrosinase inhibitors is essential for use in the cosmetic, food, medical and agricultural industries [7, 8].

Quinazolinone is a common structural motif with the fused heterocyclic system that is ubiquitous in several pharmaceuticals and natural products. Quinazolin-4(3*H*)-one exhibited varied ranges of biological properties such as antibacterial, antifungal, anti-inflammatory, anticonvulsant, anticancer, anti-HIV and analgesic activities [9–11]. In a recent effort to look for the possible application of quinazolin-4(3*H*)-one as a tyrosinase inhibitor, 2–4-fluorophenyl-quinazolin-4(3H)-one was reported as tyrosinase inhibitor with IC_50_ = 120 ± 2 μM against tyrosinase in diphenolase activity. Compound **A** was found to be a reversible and mixed-type inhibitor in kinetic studies. In silico studies revealed that this compound affected the mass transfer rate by blocking the enzyme catalytic center [12]. Oxoquinazolin-3(4H)-yl)furan-2-carboxamide (compound **B**) was found to be another potent inhibitor with an IC_50_ value of 0.028 to 1.775 µM [13]. In 2021, thio-quinazolinone (compound **C**) demonstrated good potency against tyrosinase and reduced the melanin content in the B16F10 cell line with limited toxicity [14]. Yaru Huang et al. reported the synthesis of derivative **D** with mix type inhibition pattern. Molecular docking studies showed that quinazolinone ring participated in several H-bound interactions with tyrosinase enzyme [15].

**Fig. 1:**
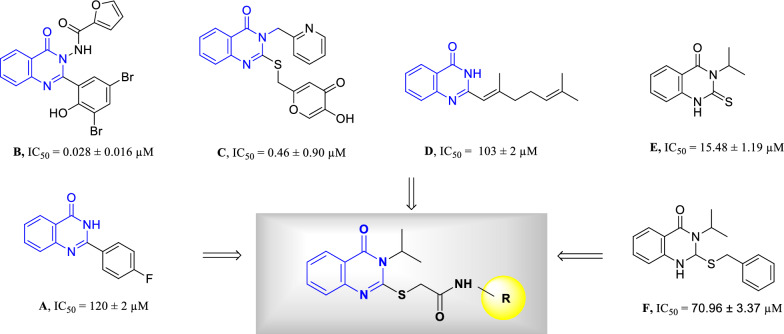
Chemical structures of some reported tyrosinase inhibitors and newly designed compound

It seems that the quinazolinone scaffold could be categorized as tyrosinase inhibitor and S, or SH groups on the designed pharmacophore, making it possible to interact with residues of the tyrosinase active site. As can be seen in compounds **B** and **C** (Fig. 1) increased bulkiness at the 3-position of quinazolinone ring improved the potencies. Recently the effects of different types of substitutions at the 3 position of quinazolinone ring were evaluated and it was shown that isopropyl moiety (compounds **E** and **F**, Fig. 1) compared to different aliphatic and aromatic moieties significantly improves the tyrosinase inhibition [16].

As result in this study, isopropyl moiety as aliphatic and bulk groups were conjugated into the 3-position of quinazolinone compared to previous studies bearing aromatic substitutions in this position. Furthermore, aryl acetamide moieties might be involved in several hydrophobic and hydrophilic interactions with the binding site via occupying the critical cavities of the enzyme. Derivatization was performed on thioquinazolinone moiety using the different aryl acetamides which could affect the tyrosinase inhibition and antioxidant potential.

Hence, the present studies illustrate the design and synthesis of new isopropylquinazolinones with various substituted analogs on acetamide moiety. After successful synthesis and characterization, all compounds were examined for their tyrosinase activity and antioxidant potential. Moreover, the kinetic and molecular docking studies of the most potent derivatives were performed.

## Results and discussion

### Chemistry

**Scheme 1:**
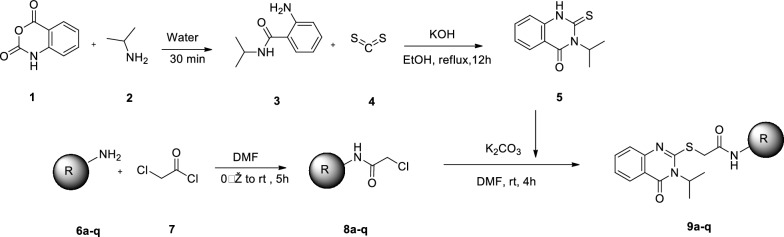
Synthesis of isopropylquinazolinone derivatives **9a–q**

A mixture of isatoic anhydride (compound **1**) and appropriate amine (compound **2**) was stirred for 30 min at room temperature in water (Scheme 1). The resulting 2-amino-N-isopropylbenzamide (compound **3**), carbon disulfide (compound **4**), and KOH in ethanol were heated under reflux for 12 h to synthesize 3-isopropyl-2-thioxo-2,3-dihydroquinazolin-4(1H)-one (compound **5**).

On the other hand, to the stiring solution of amine derivatives (compound **6a–q**) in DMF at 0 ˚C, chloroacetyl chloride (compound **7**) was added dropwise followed by stiring for 5 h at room temperature. After compeletation of the reaction, the mixture was poured into ice water to obtain, 2-chloro-N-phenylnacetamide derivatives **(8a–q)**. Finally, 3-isopropyl-2-thioxo-2,3-dihydroquinazolin-4(1H)-one (compound **5**), were added to the 2-chloro-N-phenylnacetamide derivatives (**8a–q**) in DMF in presence of potassium carbonate at room temperature for 4 h. The structure of final products **9a–q** was confirmed using NMR and IR spectroscopy as well as elemental analysis.

### Mushroom tyrosinase inhibition and structure–activity relationship

All newly synthesized compounds (**9a–q**) were examined for their inhibitory potentials against the tyrosinase enzyme and the results were presented in Table 1 and the structure–activity relationships (SARs) were constructed. From the experimental data, it appears that all of the synthesized compounds have an inhibitory effect against tyrosinase for the oxidation of L-dopa as compared with kojic acid as the positive control (IC_50_ = 23.64 ± 2.56 µM).

**Table 1:** Tyrosinase inhibitory activity of compounds **9a–q**

Compounds	R	% Inhibition at 50 µM	IC_50_ (µM)
**9a**	Phenyl	21.64 ± 3.79	NA^a^
**9b**	2-Chlorophenyl	37.97 ± 4.68	NA
**9c**	3-Chlorophenyl	27.76 ± 1.19	NA
**9d**	4-Chlorophenyl	26.86 ± 4.67	NA
**9e**	4-Bromophenyl	24.77 ± 3.12	NA
**9f**	4-Nitrophenyl	32.68 ± 5.67	NA
**9g**	2-Methylphenyl	34.62 ± 4.67	NA
**9h**	4-Methylphenyl	26.71 ± 6.42	NA
**9i**	4-Methoxyphenyl	25.37 ± 3.13	NA
**9j**	4-Hydroxyphenyl	35.48 ± 4.91	NA
**9k**	4-Ethylphenyl	20.44 ± 4.19	NA
**9l**	2,3-Dimethylphenyl	18.20 ± 5.52	NA
**9m**	2,4-Dimethylphenyl	27.31 ± 3.43	NA
**9n**	2,6-Dimethylphenyl	16.26 ± 6.57	NA
**9o**	Naphtyl	35.22 ± 3.88	NA
**9p**	Benzyl	29.08 ± 5.24	NA
**9q**	4-Fluorobenzyl	54.47 ± 3.94	34.67 ± 3.68
**Kojic acid** ^b^	-		23.64 ± 2.56

Data presented here are the mean ± S.E

^a^NA means not active

^b^Positive control

The parent compound **9a** (% inhibition at 50 µM = 21.64) exhibited weak inhibitory activity against tyrosinase in comparison with the standard kojic acid. The halogen-substituted analogs (**9b–e**) displayed improvement in the potencies compared to unsubstituted analogs. Amongst, 2-chlorophenyl (**9b**, % inhibition at 50 µM = 37.97) exhibited the best results as compared to the rest of the halogen-substituted groups. On the other hand, evaluations of *para*-halogen substituted groups did not present significant differences so that **9d** (R = 4-chlorophenyl, % inhibition at 50 µM = 26.86) and **9e** (R = 4-bromo-phenyl, % inhibition at 50 µM = 24.77) exhibited similar inhibition at 50 µM. On the other hand, **9f** with *para*-nitro moiety was recorded as the second most active derivative among the electron-withdrawing analogs which could be due to the hydrophilicity of NO_2_ group as well as its strong electron-withdrawing power.

Furthermore, in most cases, the mono electron-donating substituted analogs (**9g–k**) showed improvement in the inhibitory potential compared to the unsubstituted derivative, **9a**. The best potency among electron-donating derivatives was seen in **9j** (R: 4-hydroxyphenyl) and **9g** (R: 2-methylphenyl) with 35.48% and 34.62% inhibition at 50 µM. However, changing the position from *ortho-*methyl to *para-*methyl reduced the activity in **9h** compared to **9g**. Also, the replacement of methyl with methoxy did not improve the potency in **9i** with just 25.37% inhibition at 50 µM. The activity pattern is a bit the same in the case of the *para*-ethyl substituted compound (**9k**) which recorded 20.44% inhibition at 50 µM similar to an unsubstituted derivative. In the case of one electron-donating substitution, it was assumed that increased bulkiness or lipophilicity at the *para* position is not favorable and the exception in this trend came back to **9j** bearing *para*-hydroxyphenyl.

Compounds **9l**-**n** with dimethyl substitution mostly displayed inferior inhibitory potential compared to the unsubstituted derivative. It seems that increased steric hinder deteriorated the potencies although **9m** exhibited 27.31% inhibition at 50 µM. Noteworthy, the replacement of phenyl (**9a**) with a naphthyl ring (**9o**) resulted in improved inhibition.

Assessment of phenyl analogs compared with benzyl derivatives recorded interesting results so that derivative **9p** (R = benzyl with 29.08% inhibition at 50 µM) revealed better potencies as compared to **9a** analogs. Also, **9q** bearing 4-fluorobenzyl moiety was discovered to be the most active derivative of the series.

Overall the precise mechanism of action of the isopropylquinazolinones is unknown. However, the increase of benzyl derivatives' potencies compared to the phenyl compounds might indicate better interaction of benzyl analogs with the hydrophobic pocket of the tyrosinase enzyme and the possibility of better rotation of substitutions in the binding pocket of the enzyme. Also, SARs analysis demonstrated mostly, better inhibitory activities of *ortho*-substituted analogous (**9b** and **9g**) as compared to the *para*-substituted derivatives (**9d** and **9h**).

### Enzyme kinetic studies

**Fig. 2:**
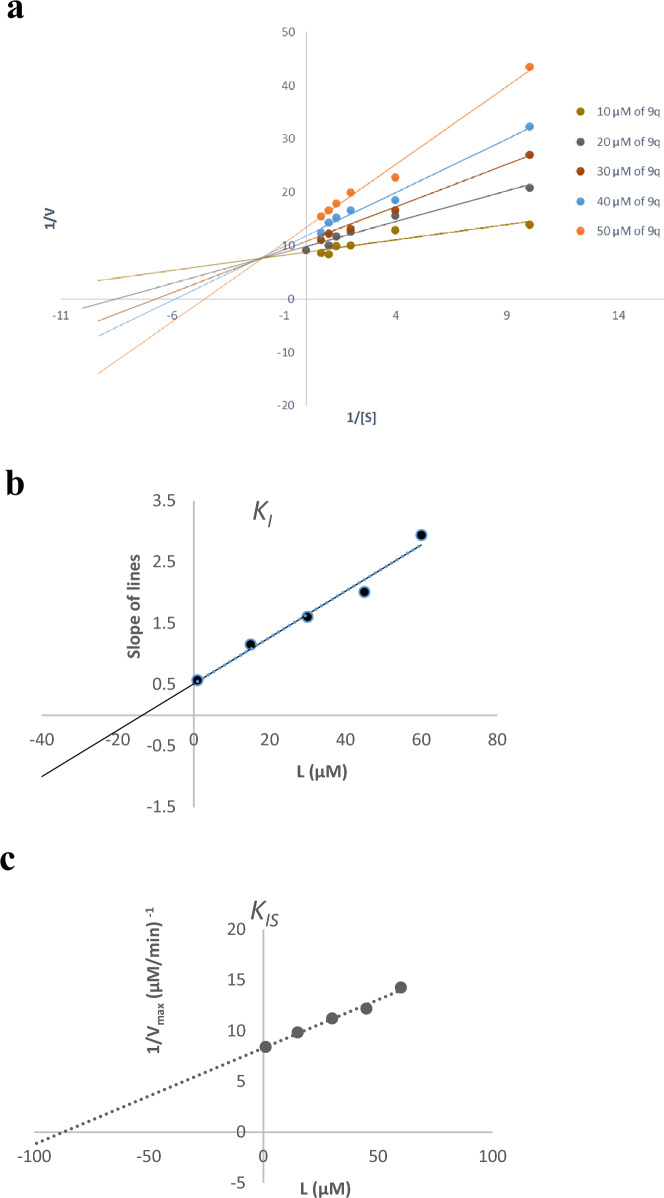
Kinetics of tyrosinase inhibition by **9q**. **a** The Lineweaver–Burk plot in the absence and presence of different concentrations of **9q**; **b** The secondary plot of slopes of the lines vs various concentrations of **9q**; **c** The secondary plot of 1/V_max_ vs various concentrations of **9q**

According to Fig. 2a, the Lineweaver–Burk plot showed that **9q** is a mix-type inhibitor in which the inhibitor may bind to the enzyme whether or not the enzyme has already bound to the substrate. Furthermore, the plot of the slope of the lines versus different concentrations of inhibitor gave an estimate of the inhibition constant (*K*_I_) of 13.60 µM (Fig. 2b). Compound **9q** also recorded the inhibition constant with the enzyme–substrate complex (*K*_*IS*_) of 87.32 µM (Fig. 2c) [17, 18].

### Inspecting the antioxidant properties of isopropylquinazolinones

In vitro antioxidant activities of the synthesized compounds were determined quantitatively using DPPH radical scavenging assay. DPPH is a stable free radical at room temperature and accepts an electron or hydrogen radical from an antioxidant to become a stable diamagnetic molecule [19]. The results of antioxidant potential are presented in Table 2. These results delineate that the unsubstituted compounds **9a** and **9p** had no antioxidant capacity while mostly any types of substitutions improved the antioxidant activities. The best potencies came back to **9o** bearing naphthyl moiety with an IC_50_ value of 38.81 µM followed by **9q** containing 4-flurobenzyl (IC_50_ = 40.73 µM). Also, 4-bromophenyl (% inhibition at 100 µM = 41.44) and 4-chloro-phenyl (% inhibition at 100 µM = 42.78) derivatives were other potent antioxidant agents. Similar to previous studies, halogens especially fluorine, increases antioxidant activity [20]. Assessments of electron donating groups showed compound **9n** bearing 2,6-dimethyl-phenyl was the most potent agent among electron donating derivatives with 44.38% inhibition at 100 µM.

**Table 2:** Antioxidant properties of synthesized compounds using DPPH assay

Compound	R	% Inhibition at 100 µM	IC_50_ (µM) ± RSD^a^
**9a**	Phenyl	2.941 ± 1.76	NA
**9b**	2-Chlorophenyl	26.47 ± 2.48	NA
**9c**	3-Chlorophenyl	18.71 ± 5.39	NA
**9d**	4-Chlorophenyl	42.78 ± 4.86	NA
**9e**	4-Bromophenyl	41.44 ± 3.85	NA
**9f**	4-Nitrophenyl	4.87 ± 1.65	NA
**9g**	2-Methylphenyl	20.71 ± 2. 71	NA
**9h**	4-Methylphenyl	29.41 ± 1.76	NA
**9i**	4-Methoxyphenyl	20.05 ± 3.47	NA
**9j**	4-Hydroxyphenyl	40.83 ± 4.53	NA
**9k**	4-Ethylphenyl	10.16 ± 4.27	NA
**9l**	2,3-Dimethylphenyl	5.08 ± 2.14	NA
**9m**	2,4-Dimethylphenyl	21.12 ± 2.99	NA
**9n**	2,6-Dimethylphenyl	44.38 ± 5.03	NA
**9o**	Naphtyl	68.44 ± 4.84	38.81 ± 3.06
**9p**	Benzyl	4.47 ± 2.35	NA
**9q**	4-Fluorobenzyl	60.33 ± 5.53	40.73 ± 2.77
**Quercetin **^**b**^		–	18.56 ± 2.19

Data presented here are the mean ± S.E of three independent experiments

^a^NA means not active

^b^Positive control

### Docking study

**Fig. 3:**
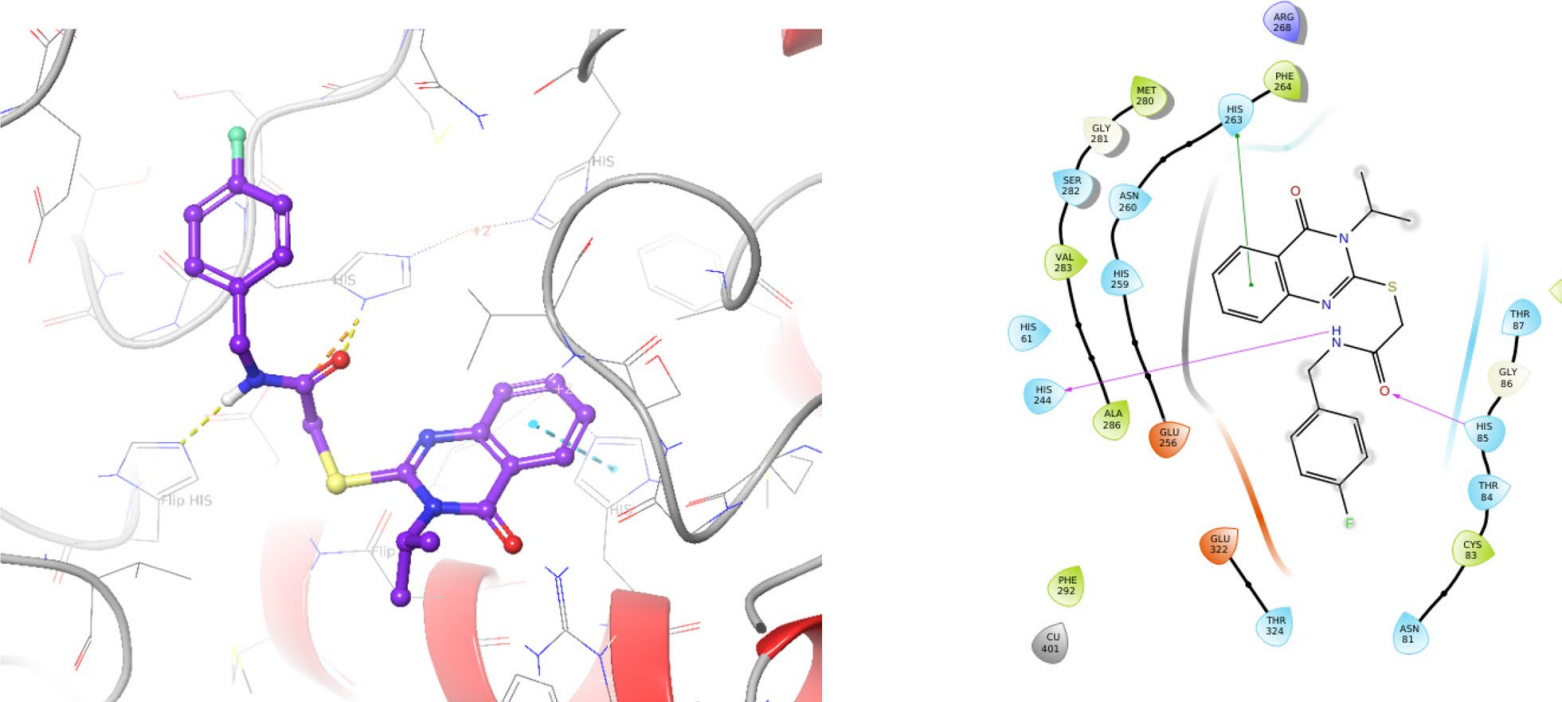
3D and 2D diagrams of compound **9q** within the binding pocket of tyrosinase

In the first step, molecular docking validation was performed with the redocking of tropolone as a native ligand inside the tyrosinase. Alignment of the best pose of tropolone in the active site of the enzyme and crystallographic ligand recorded an RMSD value of 0.42 Å (RMSD should be less than 2 Å) which confirms the accuracy of docking. Molecular docking results revealed a fit-well binding pattern of compound **9q** with the adaptation of several key interactions with active site residues (Fig. 3).

Numerous significant interactions with catalytic residues were found so that quinazolinone rings interacted through π-π staking interaction with His263 (one of the critical residues of the active site) whereas, the C=O and NH groups of acetamide moieties formed two critical hydrogen bonds with His85 and His244 of target tyrosinase protein. Literature data ensured the importance of these His residues in bonding with tyrosinase inhibitors, which strengthen our docking results.

## Conclusions

In summary, in this study, a series of isopropylquinazolinones was developed and evaluated as tyrosinase inhibitors. The SARs was presented by analyzing the impact of varying substitutions on tyrosinase inhibitory potential. The 4-fluorobenzyl-substituted compound **9q** displayed the best tyrosinase inhibition of the series. The kinetic studies of the most active compound, **9q**, showed that this compound had a mixed mode of inhibition. Also, their antioxidant properties were determined using DPPH assay and SARs analysis of antioxidant capacities showed that the *para*-halogen substituted compounds displayed better antioxidant activities. The binding conformation of the most potent derivative was determined by a molecular docking study and revealed that structural features of **9q** as an active compound play a vital role in the binding interactions of this compound with the enzyme.

## Material and methods

### Synthesis

A mixture of isatoic anhydride (compound **1**, 10 mmol) and appropriate amine (compound **2,** 10 mmol) was stirred for 30 min at room temperature in water (20 mL). After completion of the reaction (checked by TLC), the resulting white 2-amino-N-isopropylbenzamide was filtered off and used for the next reaction without further purification. 2-amino-N-isopropylbenzamide (compound **3**, 1 mmol), carbon disulfide (compound **4**, 1.2 mmol), and KOH (1.5 mmol) in ethanol (5 mL) were heated under reflux for 12 h. After completion of the reaction (checked by TLC), the reaction mixture was poured into cold water which was acidifed with HCL 6N (pH = 5). The precipitates were filtered off, and washed with cold water to obtain the pure 3-isopropyl-2-thioxo-2,3-dihydroquinazolin-4(1H)-one (compound **5**). To a solution of amine derivatives (compound **6a–q**, 1 mmol) in DMF (4 mL) at 0 °C, chloroacetyl chloride (compound **7**), was added. The mixture was stirred at room temperature for 5 h and poured into ice water and then filtered to get the obtained solids (**8a–q**) were then filtered, dried, and recrystallized from ethanol.

A mixture of 3-isopropyl-2-thioxo-2,3-dihydroquinazolin-4(1H)-one (compound **5**, 1 mmol), 2-chloro-N-phenylnacetamide derivatives (**8a–q**, 1 mmol) and, potassium carbonate (1.5 mmol) in DMF (5 ml) was stirred at rt for 4 h. Next, the mixture reaction was poured in cold water and the residue was filtrated and purified using recrystallization from ethanol to give 2-((3-isopropyl-4-oxo-3,4-dihydroquinazolin-2-yl)thio)-N-phenylacetamide derivatives (**9a–q**).

Analytical HPLC evaluation of **9b**, **9o**, and **9q** was performed on a Kanuer HPLC system equipped with UV detectors using an RP column (C18, 5 μm, 150 × 4.6 mm) and solvent acetonitrile and water with flow rate, 1 mL/min; detection, 254 nm; injection volume, 10 μL (Additional file 1: Figs. S1, S2 and S3).

### 2-((3-Isopropyl-4-oxo-3,4-dihydroquinazolin-2-yl)thio)-N-phenylacetamide (9a)

Cream solid; Yield:77%; MP = 190–192 °C; IR (KBr, v_max_) 3252 (NH), 3025 (CH Aromatic), 2970(CH Aliphatic), 1660 (C=O) Cm^−1^; ^1^H NMR (500 MHz, DMSO-*d*_*6*_) δ 10.39 (s,1H, NH _Amide_), 8.01 (d, *J* = *7.90 Hz*, 1H, H_5_), 7.71 (t, *J* = *7.60 Hz*, 1H, H_7_), 7.61 (d, *J* = *8.10 Hz*, 2H, H_2_,, H_6_,), 7.42 (d, *J* = *8.10 Hz*, 1H, H_8_), 7.38 (t, *J* = 7.50 Hz, 1H, H_6_), 7.29 (t, *J* = 7.80 Hz, 2H, H_3_, H_5_), 7.03 (t, *J* = 7.30 Hz, 1H, H_4_), 4.75–4.40 (m, 1H, CH _Isopropyl_), 4.17 (s, 2H, CH_2_), 1.59 (d, *J* = *6.70 Hz*, 6H, 2xCH_3_) ppm. ^13^C NMR (125 MHz, DMSO-d6): δ 166.2, 161.3, 156.6, 146.6, 139.5, 134.9, 129.2, 126.6, 126.3, 125.8, 123.8, 120.3, 119.5; ESI–MS (C_19_H_19_N_3_O_2_S): calculated m/z 353.12 [M + H]^+^, observed m/z 353.19[M + H]^+^; Anal. Calcd: C_19_H_19_N_3_O_2_S; C, 64.57; H, 5.42; N, 11.89; Found; C, 64.69; H, 5.57; N, 12.02.

### N-(2-chlorophenyl)-2-((3-isopropyl-4-oxo-3,4-dihydroquinazolin-2-yl)thio)acetamide (9b)

Brown solid; Yield:78%; MP = 207–209 °C; IR (KBr, v_max_) 3357 (NH), 3070 (CH Aromatic), 2980 (CH Aliphatic), 1677 (C=O) Cm^−1^; ^1^H NMR (500 MHz, DMSO-*d*_*6*_) δ 9.86 (s,1H, NH _Amide_), 8.03 (d, *J* = *7.90 Hz*, 1H, H_5_), 7.80–7.69 (m, 2H, H_7_, H_6_), 7.50 (d, *J* = *8.10 Hz*, 1H, H_8_), 7.45 (d, *J* = *8.00 Hz*, 1H, H_3_), 7.39 (t, *J* = *7.50 Hz*, 1H, H_6_), 7.28 (t, *J* = *7.70 Hz*, 1H, H_5_), 7.14 (t, *J* = *7.60 Hz*, 1H, H_4_),4.83–4.54 (m, 1H, CH _Isopropyl_), 4.27 (s, 2H, CH_2_), 1.59 (d, *J* = *6.50 Hz*, 6H, 2xCH_3_) ppm. ^13^C NMR (125 MHz, DMSO-*d*_*6*_): δ 166.8, 161.3, 156.4, 146.6,135.2, 134.8, 129.9, 127.9, 126.6, 126.3, 126.2, 126.0, 125.8, 120.3, 37.0, 19.6; ESI–MS (C_19_H_18_ClN_3_O_2_S): calculated m/z 387.08 [M + H]^+^, observed m/z 387.13 [M + H]^+^; Anal. Calcd: C_19_H_18_ClN_3_O_2_S; C, 58.83; H, 4.68; N, 10.83; Found; C, 59.13; H, 4.85; N, 11.01.

### N-(3-chlorophenyl)-2-((3-isopropyl-4-oxo-3,4-dihydroquinazolin-2-yl)thio)acetamide (9c)

Brown solid; Yield:74%; MP = 203–205 °C; IR (KBr, v_max_) 3363 (NH), 3070 (CH Aromatic), 2980 (CH Aliphatic), 1681 (C=O) Cm^−1^; ^1^H NMR (500 MHz, DMSO-*d*_*6*_) δ 10.60 (s,1H, NH _Amide_), 8.01 (d, *J* = *8.20 Hz*, 1H, H_5_), 7.81 (s, 1H, H_2_), 7.71 (t, *J* = *7.60 Hz*, 1H, H_7_), 7.48 (d, *J* = *8.50 Hz*, 1H, H_8_),7.40–7.36 (m, 2H, H_6_, H_6_), 7.34 (t, *J* = *8.00 Hz*, 1H, H_5_), 7.10 (d, *J* = *8.50 Hz*, 1H, H_4_), 4.74–4.54 (m, 1H, CH _Isopropyl_), 4.18 (s, 2H, CH_2_), 1.59 (d, *J* = *6.50 Hz*, 6H, 2xCH_3_) ppm. ^13^C NMR (125 MHz, DMSO-*d*_*6*_): δ 166.7, 161.3, 156.5, 146.6, 140.9, 134.9, 133.5, 130.9, 126.6, 126.3, 125.8, 123.5, 120.3, 119.0, 117.9, 37.7, 19.6; ESI–MS (C_19_H_18_ClN_3_O_2_S): calculated m/z 387.08 [M + H]^+^, observed m/z 387.17 [M + H]^+^; Anal. Calcd: C_19_H_18_ClN_3_O_2_S; C, 58.83; H, 4.68; N, 10.83; Found; C, 59.03; H, 4.81; N, 10.98.

### N-(4-chlorophenyl)-2-((3-isopropyl-4-oxo-3,4-dihydroquinazolin-2-yl)thio)acetamide (9d)

Brown solid; Yield:79%; MP = 211–213 °C; IR (KBr, v_max_) 3378 (NH), 3065 (CH Aromatic), 2990 (CH Aliphatic), 1703 (C=O) Cm^−1^; ^1^H NMR (500 MHz, DMSO-*d*_*6*_) δ 10.54 (s,1H, NH _Amide_), 8.01 (d, *J* = *8.00 Hz*, 1H, H_5_), 7.70 (t, *J* = *7.60 Hz*, 1H, H_7_), 7.64 (d, *J* = *8.50 Hz*, 2H, H_2_, H_6_),7.41–7.36 (m, 2H, H_8_, H_6_), 7.35 (d, *J* = *8.50 Hz*, 2H, H_3_, H_5_), 4.81–4.54 (m, 1H, CH _Isopropyl_), 4.17 (s, 2H, CH_2_), 1.58 (d, *J* = *6.60 Hz*, 6H, 2xCH_3_) ppm. ^13^C NMR (125 MHz, DMSO-*d*_*6*_): δ 166.4, 161.3, 156.5, 146.6, 138.4, 134.9, 129.1, 127.4, 126.6, 126.3, 125.8, 121.0, 120.3, 37.7, 19.6; ESI–MS (C_19_H_18_ClN_3_O_2_S): calculated m/z 387.08 [M + H]^+^, observed m/z 387.21 [M + H]^+^; Anal. Calcd: C_19_H_18_ClN_3_O_2_S; C, 58.83; H, 4.68; N, 10.83; Found; C, 59.11; H, 4.89; N, 10.98.

### N-(4-bromophenyl)-2-((3-isopropyl-4-oxo-3,4-dihydroquinazolin-2-yl)thio)acetamide (9e)

Brown solid; Yield: 84%; MP = 203–205 °C; IR (KBr, v_max_) 3378 (NH), 3020 (CH Aromatic), 2885 (CH Aliphatic), 1661 (C=O) Cm^−1^; ^1^H NMR (500 MHz, DMSO-*d*_*6*_) δ 10.54 (s,1H, NH _Amide_), 8.01 (d, *J* = *7.90 Hz*, 1H, H_5_), 7.70 (t, *J* = *7.60 Hz*, 1H, H_7_), 7.59 (d, *J* = *8.10 Hz*, 2H, H_2_, H_6_), 7.49–7.44 (m, 2H, H_8_, H_6_), 7.40–7.34 (m, 2H, H_3_, H_5_), 4.75–4.52 (m, 1H, CH _Isopropyl_), 4.17 (s, 2H, CH_2_), 1.58 (d, *J* = *6.70 Hz*, 6H, 2xCH_3_) ppm. ^13^C NMR (125 MHz, DMSO-*d*_*6*_): δ 166.5, 161.3, 156.5, 146.6, 138.8, 134.9, 132.0, 126.6, 126.3, 125.8, 121.4, 120.3, 115.4, 37.8, 19.6; ESI–MS (C_19_H_18_BrN_3_O_2_S): calculated m/z 431.03 [M + H]^+^, observed m/z 431.09 [M + H]^+^; Anal. Calcd: C_19_H_18_BrN_3_O_2_S; C, 52.78; H, 4.20; N, 9.72; Found; C, 52.91; H, 4.40; N, 9.97.

### 2-((3-isopropyl-4-oxo-3,4-dihydroquinazolin-2-yl)thio)-N-(4-nitrophenyl)acetamide (9f)

Brown solid; Yield:81%; MP = 216–218 °C; IR (KBr, v_max_) 3343(NH), 3040(CH Aromatic), 2980(CH Aliphatic), 1674(C=O), 1560–1355(NO_2)_ Cm^−1^; ^1^H NMR (500 MHz, DMSO-*d*_*6*_) δ 11.03 (s,1H, NH _Amide_), 8.22 (d, *J* = *7.50 Hz*, 2H, H_3_, H_5_), 8.00 (d, *J* = *7.90 Hz*, 1H, H_5_), 7.87 (d, *J* = *8.10 Hz*, 2H, H_2_, H_6_), 7.68 (t, *J* = *7.60 Hz*, 1H, H_7_), 7.37 (t, *J* = *7.20 Hz*, 1H, H_6_), 7.37 (d, *J* = *7.20 Hz*, 1H, H_8_), 4.84–4.43 (m, 1H, CH _Isopropyl_), 4.22 (s, 2H, CH_2_), 1.60 (d, *J* = *6.10 Hz*, 6H, 2xCH_3_) ppm. ^13^C NMR (125 MHz, DMSO-*d*_*6*_): δ 167.5, 161.3, 156.4, 146.5, 145.6, 142.7, 135.0, 126.6, 126.3, 125.7, 125. 5, 125.4, 119.2, 37.8, 19.6; ESI–MS (C_19_H_18_N_4_O_4_S): calculated m/z 398.10 [M + H]^+^, observed m/z 398.28 [M + H]^+^; Anal. Calcd: C_19_H_18_N_4_O_4_S; C, 57.27; H, 4.55; N, 14.06; Found; C, 57.43; H, 4.81; N, 14.25.

### 2-((3-isopropyl-4-oxo-3,4-dihydroquinazolin-2-yl)thio)-N-(o-tolyl)acetamide (9g)

Cream solid; Yield:76%; MP = 196–198 °C; IR (KBr, v_max_) 3238 (NH), 3035 (CH Aromatic), 2965 (CH Aliphatic), 1682 (C=O) Cm^−1^; ^1^H NMR (500 MHz, DMSO-*d*_*6*_) δ 9.69 (s,1H, NH _Amide_), 8.04 (d, *J* = *7.90 Hz*, 1H, H_5_), 7.75 (t, *J* = *7.60 Hz*, 1H, H_7_), 7.50 (d, *J* = *8.10 Hz*, 1H, H_8_), 7.41 (t, *J* = *7.50 Hz*, 1H, H_6_), 7.36 (d, *J* = *7.80 Hz*, 1H, H_3_), 7.18 (d, *J* = *7.30 Hz*, 1H, H_6_), 7.13 (t, *J* = *7.50 Hz*, 1H, H_5_), 7.06 (t, *J* = *7.30 Hz*, 1H, H_4_), 4.81–4.57 (m, 1H, CH _Isopropyl_), 4.22 (s, 2H, CH_2_), 2.18 (s, 3H, CH_3_), 1.59 (d, *J* = *6.50 Hz*, 6H, 2xCH_3_) ppm. ^13^C NMR (125 MHz, DMSO-*d*_*6*_): δ 166.2, 161.3, 156.6, 146.6, 136.6, 134.9, 132.3, 130.7, 126.6, 126.4, 126.3, 125.9, 125.8, 125.4, 120.4, 34.0, 19.6, 18.2; ESI–MS (C_20_H_21_N_3_O_2_S): calculated m/z 387.08 [M + H]^+^, observed m/z 387.13 [M + H]^+^; Anal. Calcd: C_20_H_21_N_3_O_2_S; C, 65.37; H, 5.76; N, 11.44; Found; C, 65.52; H, 5.91; N, 11.67.

### 2-((3-isopropyl-4-oxo-3,4-dihydroquinazolin-2-yl)thio)-N-(p-tolyl)acetamide (9h)

Cream solid; Yield:76%; MP = 199–201 °C; IR (KBr, v_max_) 3400 (NH), 3020(CH Aromatic), 2975(CH Aliphatic) 1701(C=O) Cm^−1^; ^1^H NMR (500 MHz, DMSO-*d*_*6*_) δ 10.44 (s,1H, NH _Amide_), 8.01 (d, *J* = *7.90 Hz*, 1H, H_5_), 7.71 (t, *J* = *7.60 Hz*, 1H, H_7_), 7.50 (d, *J* = *8.30 Hz*, 2H, H_2_, H_6_), 7.42 (d, *J* = *8.10 Hz*, 1H, H_8_), 7.38 (t, *J* = *7.50 Hz*, 1H, H_6_), 7.09 (d, *J* = *8.10 Hz*, 2H, H_3_, H_5_), 4.75–4.57 (m, 1H, CH _Isopropyl_), 4.17 (s, 2H, CH_2_), 2.22 (s, 3H, CH_3_), 1.59 (d, *J* = *6.60 Hz*, 6H, 2xCH_3_) ppm. ^13^C NMR (125 MHz, DMSO-*d*_*6*_): δ 166.0, 161.3, 156.6, 146.6, 137.0, 134.9, 132.7, 129.5, 126.5, 126.2, 125.9, 120.3, 119.5, 37.7, 20.8, 19.6; ESI–MS (C_20_H_21_N_3_O_2_S): calculated m/z 367.14 [M + H]^+^, observed m/z 367.28 [M + H]^+^; Anal. Calcd: C_20_H_21_N_3_O_2_S; C, 65.37; H, 5.76; N, 11.44; Found; C, 65.51; H, 5.97; N, 11.59.

### 2-((3-isopropyl-4-oxo-3,4-dihydroquinazolin-2-yl)thio)-N-(4-methoxyphenyl)acetamide (9i)

Cream solid; Yield:88%; MP = 205–207 °C; IR (KBr, v_max_) 3253 (NH), 3030 (CH Aromatic), 2975(CH Aliphatic), 1661(C=O) Cm^−1^; ^1^H NMR (500 MHz, DMSO-*d*_*6*_) δ 10.25 (s,1H, NH _Amide_), 8.02 (d, *J* = *7.90 Hz*, 1H, H_5_), 7.72 (t, *J* = *7.60 Hz*, 1H, H_7_), 7.51 (d, *J* = *8.20 Hz*, 2H, H_2_, H_6_),7.44 (d, *J* = *8.10 Hz*, 1H, H_8_), 7.39 (t, *J* = *7.60 Hz*, 1H, H_6_), 6.87 (d, *J* = *8.20 Hz*, 2H, H_3_, H_5_), 4.83–4.51 (m, 1H, CH _Isopropyl_), 4.14 (s, 2H, CH_2_), 3.70 (s, 3H, CH_3_), 1.59 (d, *J* = *6.50 Hz*, 6H, 2xCH_3_) ppm. ^13^C NMR (125 MHz, DMSO-*d*_*6*_): δ 165.7, 161.3, 156.6, 155.7, 146.6, 134.9, 132.6, 126.6, 126.3, 125.9, 121.1, 120.3, 114.3, 55.5, 37.7, 19.6; ESI–MS (C_20_H_21_N_3_O_3_S): calculated m/z 383.13 [M + H]^+^, observed m/z 383.29 [M + H]^+^; Anal. Calcd: C_20_H_21_N_3_O_3_S; C, 62.64; H, 5.52; N, 10.96; Found; C, 62.82; H, 5.75; N, 11.13.

### N-(4-hydroxyphenyl)-2-((3-isopropyl-4-oxo-3,4-dihydroquinazolin-2-yl)thio)acetamide(9j)

Cream solid; Yield:77%; MP = 193–195 °C; IR (KBr, v_max_) 3295 (NH), 3075(CH Aromatic), 2970(CH Aliphatic), 1675 (C=O) Cm^−1^; ^1^H NMR (500 MHz, DMSO-*d*_*6*_) δ 11.98 (s,1H, OH), 10.25 (s,1H, NH _Amide_), 8.03 (d, *J* = *7.70 Hz*, 1H, H_5_), 7.74 (t, *J* = *7.50 Hz*, 1H, H_7_), 7.52(d, *J* = *8.00 Hz*, 2H, H_2_, H_6_),7.43 (d, *J* = *7.90 Hz*, 1H, H_8_), 7.39 (t, *J* = *7.50 Hz*, 1H, H_6_), 6.88 (d, *J* = *8.10 Hz*, 2H, H_3_, H_5_), 4.67 (m, 1H, CH_Isopropyl_), 4.25 (s, 2H, CH_2_), 1.60 (d, *J* = *6.50 Hz*, 3H, CH_3_) 1.59 (d, *J* = *6.40 Hz*, 3H, CH_3_) ppm. ^13^C NMR (125 MHz, DMSO-*d*_*6*_): δ 165.5, 161.4, 156.7, 155.8, 147.6, 134.9, 132.6, 127.6, 127.3, 126.8, 122.1, 120.5, 115.4, 38.7, 19.6; ESI–MS (C_19_H_19_N_3_O_3_S): calculated m/z 369.11 [M + H]^+^, observed m/z 370.11 [M + H]^+^; Anal. Calcd: C_19_H_19_N_3_O_3_S; C, 61.77; H, 5.18; N, 11.37; Found; C, 62.05; H, 5.81; N, 11.17.

### N-(4-ethylphenyl)-2-((3-isopropyl-4-oxo-3,4-dihydroquinazolin-2-yl)thio)acetamide (9k)

Cream solid; Yield:81%; MP = 195–197 °C; IR (KBr, v_max_) 3401 (NH), 3065(CH Aromatic), 2950(CH Aliphatic), 1670 (C=O) Cm^−1^; ^1^H NMR (500 MHz, DMSO-*d*_*6*_) δ 10.44 (s,1H, NH _Amide_), 8.01 (d, *J* = *7.40 Hz*, 1H, H_5_), 7.72 (t, *J* = *7.60 Hz*, 1H, H_7_), 7.52 (d, *J* = *8.30 Hz*, 2H, H_2_, H_6_), 7.43 (d, *J* = *8.20 Hz*, 1H, H_8_), 7.39 (t, *J* = *7.60 Hz*, 1H, H_6_), 7.12 (d, *J* = *8.10 Hz*, 2H, H_3_, H_5_), 4.76–4.59 (m, 1H, CH _Isopropyl_), 4.17 (s, 2H, CH_2_), 2.52 (q, 2H, CH_2 Ethyl_), 1.58 (d, *J* = *6.60 Hz*, 6H, 2xCH_3_), 1.13 (t, 3H, CH_3 Ethyl_) ppm. ^13^C NMR (125 MHz, DMSO-*d*_*6*_): δ 166.0, 161.3, 156.6, 146.6, 139.1, 137.2, 134.9, 128.3, 126.5, 126.3, 125.9, 120.3, 119.6, 37.7, 28.0, 19.6, 16.1. ESI–MS (C_21_H_23_N_3_O_2_S): calculated m/z 381.15 [M + H]^+^, observed m/z 381.27 [M + H]^+^; Anal. Calcd: C_21_H_23_N_3_O_2_S; C, 66.12; H, 6.08; N, 11.01; Found C, 66.37; H, 6.24; N, 11.35.

### N-(2,3-dimethylphenyl)-2-((3-isopropyl-4-oxo-3,4-dihydroquinazolin-2-yl)thio)acetamide (9l)

Brown solid; Yield:69%; MP = 217–219 °C; IR (KBr, v_max_) 3371 (NH), 3040 (CH Aromatic), 2985 (CH Aliphatic), 1669 (C=O) Cm^−1^; ^1^H NMR (500 MHz, DMSO-*d*_*6*_) δ 9.86 (s, 1H, NH _Amide_), 8.03 (d, *J* = *7.90 Hz*, 1H, H_5_), 7.75 (t, *J* = *7.20 Hz*, 1H, H_7_), 7.51 (d, *J* = *8.20 Hz*, 1H, H_8_), 7.41 (t, *J* = *7.50 Hz*, 1H, H_6_), 7.09 (d, *J* = *7.50 Hz*, 1H, H_6_),7.03–6.95 (m, 2H, H_4_, H_5_), 4.75–4.61 (m, 1H, CH _Isopropyl_), 4.21 (s, 2H, CH_2_), 2.20 (s, 3H, CH_3_), 2.04 (s, 3H, CH_3_), 1.59 (d, *J* = *6.60 Hz*, 6H, 2xCH_3_) ppm. ^13^C NMR (125 MHz, DMSO-*d*_*6*_): δ 166.2, 161.4, 156.6, 146.7, 137.4, 136.4, 134.9, 131.8, 127.5, 126.6, 126.3, 126.0, 125.6, 123.9, 120.3, 37.0, 20.5, 19.6, 14.5; ESI–MS (C_21_H_23_N_3_O_2_S): calculated m/z 381.15 [M + H]^+^, observed m/z 381.24 [M + H]^+^; Anal. Calcd: C_21_H_23_N_3_O_2_S; C, 66.12; H, 6.08; N, 11.01; Found; C, 66.33; H, 6.27; N, 11.21.

### N-(2,4-dimethylphenyl)-2-((3-isopropyl-4-oxo-3,4-dihydroquinazolin-2-yl)thio)acetamide (9m)

Brown solid; Yield: 69%; MP = 222–225 °C; IR (KBr, v_max_) 3376 (NH), 3045 (CH Aromatic), 2995 (CH Aliphatic), 1674 (C=O) Cm^−1^; ^1^H NMR (500 MHz, DMSO-*d*_*6*_) δ 9.57 (s,1H, NH _Amide_), 8.04 (d, *J* = *7.90 Hz*, 1H, H_5_), 7.77 (t, *J* = *7.60 Hz*, 1H, H_7_), 7.53 (d, *J* = *8.80 Hz*, 1H, H_6_), 7.42 (t, *J* = *7.50 Hz*, 1H, H_6_), 7.09–6.98 (m, 3H, H_8_, H_3_, H_5_), 4.81–4.60 (m, 1H, CH _Isopropyl_), 4.23 (s, 2H, CH_2_), 3.32 (s, 3H, CH_3_), 2.09 (s, 3H, CH_3_), 1.60 (d, *J* = *6.60 Hz*, 6H, 2xCH_3_) ppm. ^13^C NMR (125 MHz, DMSO-*d*_*6*_): δ 165.7, 161.4, 156.5, 146.7, 135.7, 135.3, 134.9, 128.0, 127.0, 126.6, 126.3, 126.0, 34.4, 19.6, 18.5; ESI–MS (C_21_H_23_N_3_O_2_S): calculated m/z 381.15 [M + H]^+^, observed m/z 381.31 [M + H]^+^; Anal. Calcd: C_21_H_23_N_3_O_2_S; C, 66.12; H, 6.08; N, 11.01; Found; C, 66.28; H, 6.34; N, 11.17.

### N-(2,6-dimethylphenyl)-2-((3-isopropyl-4-oxo-3,4-dihydroquinazolin-2-yl)thio)acetamide (9n)

Cream solid; Yield:68%; MP = 215–217 °C; IR (KBr, v_max_) 3235 (NH), 3030 (CH Aromatic), 2970 (CH Aliphatic), 1684 (C=O) Cm^−1^; ^1^H NMR (500 MHz,DMSO-*d*_*6*_) δ 9.57 (s,1H, NH _Amide_), 8.04 (d, *J* = *8.00 Hz*, 1H, H_5_), 7.77 (t, *J* = *7.70 Hz*, 1H, H_7_), 7.53 (d, *J* = *8.20 Hz*, 1H, H_8_), 7.42 (t, *J* = *7.50 Hz*, 1H, H_6_), 7.05–7.00 (m,3H, H_4_, H_3_, H_5_), 4.79–4.64 (m, 1H, CH_Isopropyl_), 4.23 (s, 2H, CH_2_), 2.09 (s, 6H, 2 × CH_3_), 1.60 (d, *J* = *6.60 Hz*, 6H, 2xCH_3_) ppm. ^13^C NMR (125 MHz, DMSO-*d*_*6*_): δ 165.7, 161.4, 156.5,146.7, 135.7, 135.3, 134.9,128.0, 127.0, 126.6, 126.3, 126.0, 120.4, 36.4, 19.6, 18.5; ESI–MS (C_21_H_23_N_3_O_2_S): calculated m/z 381.15 [M + H]^+^, observed m/z 381.24 [M + H]^+^; Anal. Calcd: C_21_H_23_N_3_O_2_S; C, 66.12; H, 6.08; N, 11.01; Found; C, 66.23; H, 6.29; N, 11.26.

### 2-((3-isopropyl-4-oxo-3,4-dihydroquinazolin-2-yl)thio)-N-(naphthalen-2-yl)acetamide (9o)

Cream solid; Yield:65%; MP = 217–219 °C; IR (KBr, v_max_) 3291 (NH), 3065 (CH Aromatic), 2980(CH Aliphatic), 1675 (C=O) Cm^−1^; ^1^H NMR (500 MHz, DMSO-*d*_*6*_) δ 10.36 (s,1H, NH_Amide_), 8.17 (d, *J* = *7.8 Hz*, 1H, H_8_), 8.05 (d, *J* = *7.80 Hz*, 1H, H_5_), 7.86 (t, *J* = *7.60 Hz*, 1H, H_7_), 7.84–7.80 (m, 1H, H_8_), 7.75 (t, *J* = 7.50 Hz, 1H, H_6_), 7.64 (d, *J* = 7.80 Hz, 1H, H_7_), 7.61–7.56 (m, *2*H, H_3_, H_6_), 7.46 (t, *J* = 7.50 Hz, 1H, H_4_), 7.13 (t, *J* = 7.60 Hz, 1H, H_5_), 4.25–4.12 (m, 1H, CH _Isopropyl_), 4.01 (s, 2H, CH_2_), 0.80 (d, *J* = *6.70 Hz*, 6H, 2xCH_3_) ppm. ^13^C NMR (125 MHz, DMSO-*d*_*6*_): δ 165.9, 164.2, 161.2, 156.8, 147.7, 135.8, 135.5, 134.5, 132.2, 132.0, 131.2, 128.8, 127.0, 126.4, 126.3, 121.2, 121.2, 115.9, 115.7, 37.4, 13.7; ESI–MS (C_23_H_21_N_3_O_2_S): calculated m/z 403.14 [M + H]^+^, observed m/z 403.27 [M + H]^+^; Anal. Calcd: C_23_H_21_N_3_O_2_S; C, 68.46; H, 5.25; N, 10.41; Found; C, 68.65; H, 5.42; N, 10.60.

### N-benzyl-2-((3-isopropyl-4-oxo-3,4-dihydroquinazolin-2-yl)thio)acetamide (9p)

Cream solid; Yield:81%; MP = 194–196 °C; IR (KBr, v_max_) 3264 (NH), 3025(CH Aromatic), 2960 (CH Aliphatic),1662(C=O) Cm^−1^; ^1^H NMR (500 MHz, DMSO-*d*_*6*_) δ 8.73 (t, *J* = *6.20 Hz*, 1H, NH _Amide_), 8.03 (d, *J* = *8.10 Hz*, 1H, H_5_), 7.73 (t, *J* = *7.60 Hz*, 1H, H_7_), 7.45–7.38 (m, 2H, H_8_, H_6_), 7.24–7.18 (m, 5H, H _Benzyl_), 4.77–4.56 (m, 1H, CH _Isopropyl_), 4.32 (d, *J* = *5.90 Hz*, 2H, CH_2 Benzyl_), 4.05 (s, 2H, CH_2_), 1.58 (d, *J* = *6.50 Hz*, 6H, 2xCH_3_) ppm. ^13^C NMR (125 MHz, DMSO-d6): δ 167.2, 161.4, 156.5, 146.6, 139.6, 134.8, 128.6, 127.5, 127.5, 127.1, 126.5, 126.2, 126.1, 120.3, 43.0, 36.5, 19.6; ESI–MS (C_20_H_21_N_3_O_2_S): calculated m/z 367.14 [M + H]^+^, observed m/z 367.26 [M + H]^+^; Anal. Calcd: C_20_H_21_N_3_O_2_S; C, 65.37; H, 5.76; N, 11.44; Found; C, 65.49; H, 5.98; N, 11.63.

### N-(4-fluorobenzyl)-2-((3-isopropyl-4-oxo-3,4-dihydroquinazolin-2-yl)thio)acetamide (9q)

Cream solid; Yield:76%; MP = 224–226 °C; IR (KBr, v_max_) 3314 (NH), 3050(CH Aromatic), 2960(CHAliphatic), 1671(C=O) Cm^−1^; ^1^H NMR (500 MHz, DMSO-*d*_*6*_) δ 8.83 (t, *J* = *6.10 Hz*, 1H, NH _Amide_), 8.02 (d, *J* = *8.00 Hz*, 1H, H_5_), 7.73 (t, *J* = *7.60 Hz*, 1H, H_7_), 7.41 (t, *J* = *7.50 Hz*, 1H, H_6_), 7.36 (d, *J* = *8.10 Hz*, 1H, H_8_), 7.29–7.23 (m, 2H, H_2_, H_6_), 7.04–6.97 (m, 2H, H_3_, H_5_), 4.72–4.58 (m, 1H, CH _Isopropyl_), 4.28 (d, *J* = *5.90 Hz*, 2H, CH_2 Benzyl_), 4.03 (s, 2H, CH_2_), 1.56 (d, *J* = *6.50 Hz*, 6H, 2xCH_3_) ppm. ^13^C NMR (125 MHz, DMSO-d6): δ 167.2, 162.5, 161.4, 160.5, 156.5, 146.6, 135.9, 135.8, 134.8, 129.5, 129.5, 126.5, 126.2, 126.0, 120.3, 115.3, 115.2, 42.2, 36.5, 19.6; ESI–MS (C_20_H_20_FN_3_O_2_S): calculated m/z 385.13 [M + H]^+^, observed m/z 385.26 [M + H]^+^; Anal. Calcd: C_20_H_20_FN_3_O_2_S; C, 62.32; H, 5.23; N, 10.90; Found; C, 62.54; H, 5.37; N, 11.07.

### Screening of tyrosinase inhibitory activity

Tyrosinase inhibitory activity was determined based on the previously described procedure with a slight modification [3, 21]. In brief, the test reaction mixture comprised 10 µL of each derivative, and 10 µL of mushroom tyrosinase (500 units; Sigma-Aldrich, St Louis, MO, USA) in 160 potassium phosphate buffer (pH = 6.8). The reaction mixture was incubated at 37 °C for 20 min and then 20 µL of L-Dopa (0.5 mmol/L) was added to each well, and the absorption was measured at 475 nm. The absorbance of the same mixture without tyrosinase was used as the control. Kojic acid was used as a positive control. The optical density of the inhibition in the control was considered to represent 100%. The data are expressed as mean percentages and the results were repeated in triplicate.

### Enzyme kinetic studies

The mode of inhibition of the most active compound **9q** identified with the lowest IC_50_, was investigated against tyrosinase activity with different concentrations of L-Dopa (0.1.5 mM) as substrate in the absence and presence of **9q** at different concentrations (10, 20, 30, 40 and 50 µM). A Lineweaver–Burk plot was generated to identify the type of inhibition and the Michaelis–Menten constant (*K*_m_) value was determined from the plot between the reciprocal of the substrate concentration (1/[S]) and reciprocal of enzyme rate (1/V) over various inhibitor concentrations.

### Antioxidant activities

The DPPH antioxidant potencies of all derivatives were performed according to previously published articles [22].

### Molecular docking

The molecular docking studies were performed using the Maestro Molecular Modeling platform (version 10.5) by Schrödinger, LLC. The X-ray crystal structure of the receptor (PDB ID: 2Y9X) was extracted from the PDB database. The protein is then prepared using a protein preparation wizard, the co-crystallized ligands and all water molecules were removed [23], the missing side chains and loops were filled using the prime tool, and PROPKA assigned H-bonds at pH: 7.4. To prepare the ligands, the 2D structures of the ligands were drawn in ChemDraw and converted into SDF files and subjected to ligprep module [24]. Ligands were prepared by OPLS_2005 force field using EPIK. The grid box was generated for each binding site using entries with a box size of 20 Å, the derivative was docked on binding sites using induced-fit docking, reporting 10 poses per ligand to form the final complex.

## Supplementary Information

**Additional file 1: Figure. S1**. HPLC results of 9a. **Figure S2.** HPLC results of 9n. **Figure S3**. HPLC results of 9p. **Figure S4**. 1HNMR spectrum of compound 9a. **Figure S5.** 13CNMR spectrum of compound 9a. **Figure S6**. 1HNMR spectrum of compound 9b. **Figure S7**. 13CNMR spectrum of compound 9b. **Figure S8**. 1HNMR spectrum of compound 9c. **Figure S9.** 13CNMR spectrum of compound 9c. **Figure S10**. 1HNMR spectrum of compound 9d. **Figure S11**. 13CNMR spectrum of compound 9d. **Figure S12**. 1HNMR spectrum of compound 9e. **Figure S13**. 13CNMR spectrum of compound 9e. **Figure S14**. 1HNMR spectrum of compound 9f. **Figure S15**. 13CNMR spectrum of compound 9f. **Figure S16**. 1HNMR spectrum of compound 9g. **Figure S17**. 13CNMR spectrum of compound 9g. **Figure S18**. 1HNMR spectrum of compound 9h. **Figure S19**. 13CNMR spectrum of compound 9h. **Figure S20**. 1HNMR spectrum of compound 9i. **Figure S21**. 13CNMR spectrum of compound 9i. **Figure S22**. 1HNMR spectrum of compound 9j. **Figure S23**. 13CNMR spectrum of compound 9j. **Figure S24**. 1HNMR spectrum of compound 9k. **Figure S25**. 13CNMR spectrum of compound 9k. **Figure S26**. 1HNMR spectrum of compound 9l. **Figure S27**. ^13^CNMR spectrum of compound 9l. **Figure S28**. 1HNMR spectrum of compound 9m. **Figure S29**. 13CNMR spectrum of compound 9m. **Figure S30**. 1HNMR spectrum of compound 9n. **Figure S31**. 13CNMR spectrum of compound 9n. **Figure S32**. 1HNMR spectrum of compound 9o. **Figure S33**. 13CNMR spectrum of compound 9o. **Figure S34**. 1HNMR spectrum of compound 9p. **Figure S35**. 13CNMR spectrum of compound 9p. **Figure S36**. 1HNMR spectrum of compound 9q. **Figure S37**. 13CNMR spectrum of compound 9q.

## Data Availability

The datasets generated and/or analyzed during the current study are available in the Worldwide Protein Data Bank (wwPDB) repository; (https://www.rcsb.org/structure/2y9x) with PDB https://doi.org/10.2210/pdb2Y9X/pdb.

## References

[1] Sepehri N, Iraji A, Yavari A, Asgari MS, Zamani S, Hosseini S, Bahadorikhalili S, Pirhadi S, Larijani B, Khoshneviszadeh M (2021). The natural-based optimization of kojic acid conjugated to different thio-quinazolinones as potential anti-melanogenesis agents with tyrosinase inhibitory activity. Bioorg Med Chem.

[2] Hosseinpoor H, Farid SM, Iraji A, Askari S, Edraki N, Hosseini S, Jamshidzadeh A, Larijani B, Attarroshan M, Pirhadi S (2021). Anti-melanogenesis and anti-tyrosinase properties of aryl-substituted acetamides of phenoxy methyl triazole conjugated with thiosemicarbazide: design, synthesis and biological evaluations. Bioorg Chem.

[3] Iraji A, Adelpour T, Edraki N, Khoshneviszadeh M, Miri R, Khoshneviszadeh M (2020). Synthesis, biological evaluation and molecular docking analysis of vaniline–benzylidenehydrazine hybrids as potent tyrosinase inhibitors. BMC Chem.

[4] Karimian S, Kazemi F, Attarroshan M, Gholampour M, Hemmati S, Sakhteman A, Behzadipour Y, Kabiri M, Iraji A, Khoshneviszadeh M (2021). Design, synthesis, and biological evaluation of symmetrical azine derivatives as novel tyrosinase inhibitors. BMC Chem.

[5] Karimian S, Kazemi F, Attarroshan M, Gholampour M, Hemmati S, Sakhteman A, Behzadipour Y, Kabiri M, Iraji A, Khoshneviszadeh M (2021). Design, synthesis, and biological evaluation of symmetrical azine derivatives as novel tyrosinase inhibitors. BMC Chem.

[6] Sepehri N, Khoshneviszadeh M, Farid SM, Moayedi SS, Asgari MS, Moazzam A, Hosseini S, Adibi H, Larijani B, Pirhadi S (2022). Design, synthesis, biological evaluation, and molecular docking study of thioxo-2, 3-dihydroquinazolinone derivative as tyrosinase inhibitors. J Mol Struct.

[7] Hosseinpoor H, Iraji A, Edraki N, Pirhadi S, Attarroshan M, Khoshneviszadeh M, Khoshneviszadeh M (2020). A series of benzylidenes linked to hydrazine-1-carbothioamide as tyrosinase inhibitors: synthesis, biological evaluation and structure–activity relationship. Chem Biodivers.

[8] Iraji A, Sheikhi N, Attarroshan M, Ardani GRS, Kabiri M, Bafghi AN, Kobarfard F, Rezaei Z, Khoshneviszadeh M, Foroumadi A (2022). Design, synthesis, spectroscopic characterization, in vitro tyrosinase inhibition, antioxidant evaluation, in silico and kinetic studies of substituted indole-carbohydrazides. Bioorgan Chem.

[9] FZ M, HH M, Synthesis and biological evaluation studies of novel quinazolinone derivatives as antibacterial and anti-inflammatory agents, Saudi Pharm J SPJ. 2014; 22(2): 157–62. 10.1016/j.jsps.2013.03.004 PMC3950501 24648828

[10] Ding PP, Gao M, Mao BB, Cao SL, Liu CH, Yang CR, Li ZF, Liao J, Zhao H, Li Z, Li J, Wang H, Xu X (2016). Synthesis and biological evaluation of quinazolin-4(3H)-one derivatives bearing dithiocarbamate side chain at C2-position as potential antitumor agents. Eur J Med Chem.

[11] Mirgany TO, Abdalla AN, Arifuzzaman M, Motiur Rahman AFM, Al-Salem HS (2021). Quinazolin-4(3H)-one based potential multiple tyrosine kinase inhibitors with excellent cytotoxicity. J Enzyme Inhibit Med Chem..

[12] Wang R, Chai W-M, Yang Q, Wei M-K, Peng Y (2016). 2-(4-Fluorophenyl)-quinazolin-4(3H)-one as a novel tyrosinase inhibitor: synthesis, inhibitory activity, and mechanism. Bioorg Med Chem.

[13] Dige NC, Mahajan PG, Raza H, Hassan M, Vanjare BD, Hong H, Hwan Lee K, Latip J, Seo S-Y (2019). Ultrasound mediated efficient synthesis of new 4-oxoquinazolin-3(4H)-yl)furan-2-carboxamides as potent tyrosinase inhibitors: mechanistic approach through chemoinformatics and molecular docking studies. Bioorgan Chem.

[14] Sepehri N, Iraji A, Yavari A, Asgari MS, Zamani S, Hosseini S, Bahadorikhalili S, Pirhadi S, Larijani B, Khoshneviszadeh M, Hamedifar H, Mahdavi M, Khoshneviszadeh M (2021). The natural-based optimization of kojic acid conjugated to different thio-quinazolinones as potential anti-melanogenesis agents with tyrosinase inhibitory activity. Bioorg Med Chem.

[15] Huang Y, Yang J, Chi Y, Gong C, Yang H, Zeng F, Gao F, Hua X, Wang Z (2022). Newly designed quinazolinone derivatives as novel tyrosinase inhibitor: synthesis, inhibitory activity, and mechanism. Molecules.

[16] Sepehri N, Khoshneviszadeh M, Farid SM, Moayedi SS, Asgari MS, Moazzam A, Hosseini S, Adibi H, Larijani B, Pirhadi S, Attarroshan M, Sakhteman A, Kabiri M, Hamedifar H, Iraji A, Mahdavi M (2022). Design, synthesis, biological evaluation, and molecular docking study of thioxo-2,3-dihydroquinazolinone derivative as tyrosinase inhibitors. J Mol Struct.

[17] Cao S, Wang D, Cheng R, Shi W, Zhang Q, Zeng H, Chen J (2022). Modulation of the lipophilicity and molecular size of thiosemicarbazone inhibitors to regulate tyrosinase activity. Spectrochim Acta Part A Mol Biomol Spectrosc.

[18] Hałdys K, Goldeman W, Anger-Góra N, Rossowska J, Latajka R (2021). Monosubstituted acetophenone thiosemicarbazones as potent inhibitors of tyrosinase: synthesis, inhibitory studies, and molecular docking. Pharmaceuticals.

[19] Iraji A, Firuzi O, Khoshneviszadeh M, Nadri H, Edraki N, Miri R (2018). Synthesis and structure–activity relationship study of multi-target triazine derivatives as innovative candidates for treatment of Alzheimer's disease. Bioorg Chem.

[20] Haider K, Haider MR, Neha K, Yar MS (2020). Free radical scavengers: an overview on heterocyclic advances and medicinal prospects. Eur J Med Chem.

[21] Alizadeh N, Sayahi MH, Iraji A, Yazzaf R, Moazzam A, Mobaraki K, Adib M, Attarroshan M, Larijani B, Rastegar H (2022). Evaluating the effects of disubstituted 3-hydroxy-1H-pyrrol-2 (5H)-one analog as novel tyrosinase inhibitors. Bioorg Chem.

[22] Mardani M, Afra SM, Tanideh N, Tadbir AA, Modarresi F, Koohi-Hosseinabadi O, Iraji A, Sepehrimanesh M (2016). Hydroalcoholic extract of Carum carvi L. in oral mucositis: a clinical trial in male golden hamsters. Oral Dis.

[23] Pedrood K, Rezaei Z, Khavaninzadeh K, Larijani B, Iraji A, Hosseini S, Mojtabavi S, Dianatpour M, Rastegar H, Faramarzi MA, Hamedifar H, Hajimiri MH, Mahdavi M (2022). Design, synthesis, and molecular docking studies of diphenylquinoxaline-6-carbohydrazide hybrids as potent α-glucosidase inhibitors. BMC Chem.

[24] Zarenezhad E, Montazer MN, Tabatabaee M, Irajie C, Iraji A (2022). New solid phase methodology for the synthesis of biscoumarin derivatives: experimental and in silico approaches. BMC Chem.

