# Supplementary material for: Synthesis and tyrosinase inhibitory activities of novel isopropylquinazolinones
Source: BMC Chem. 2023 Jun 23;17(1):65. doi: 10.1186/s13065-023-00978-3 (PMC10290372; doi:10.1186/s13065-023-00978-3)

Figure. S1. HPLC results of 9a


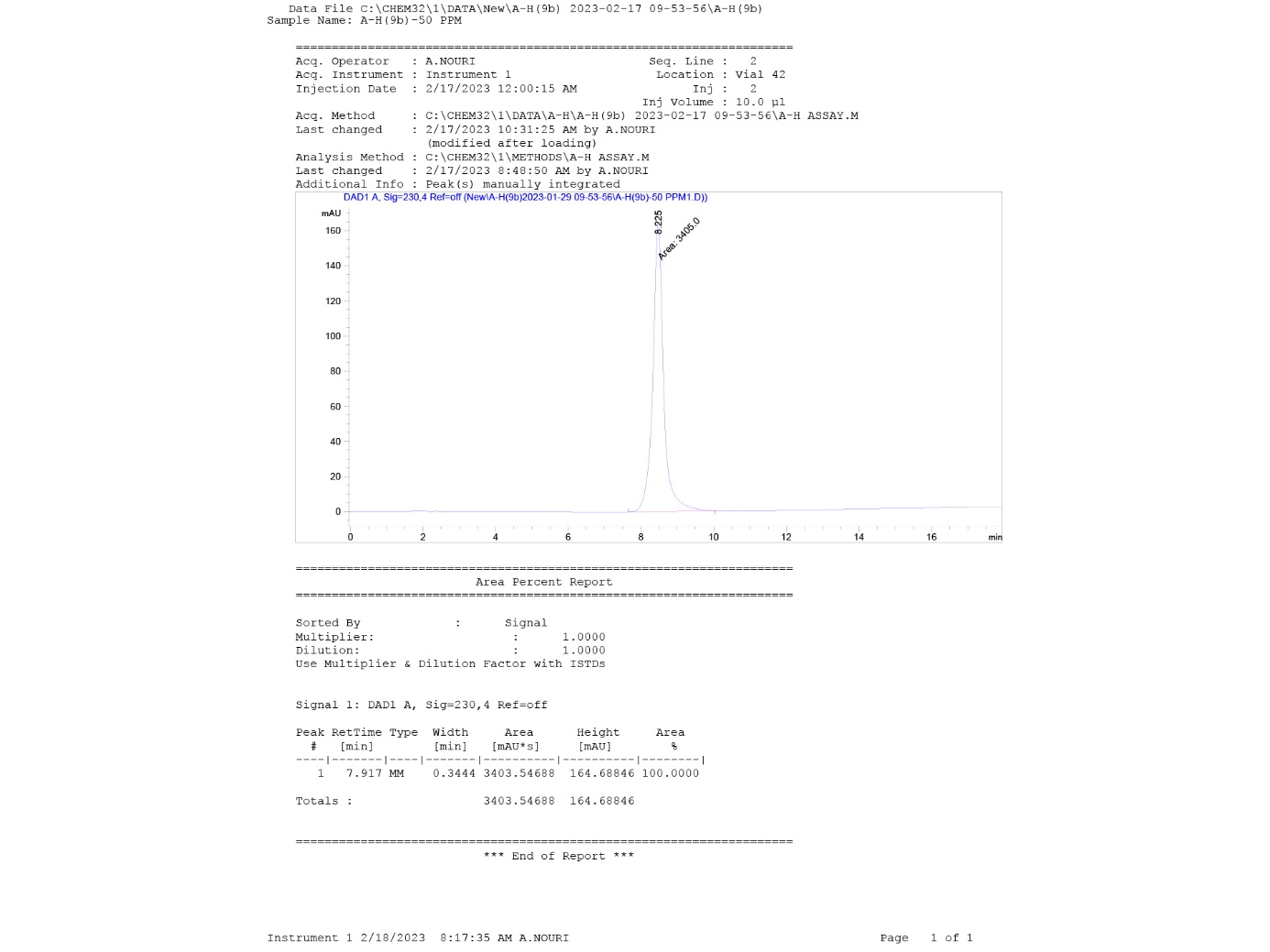


Figure. S2. HPLC results of 9n


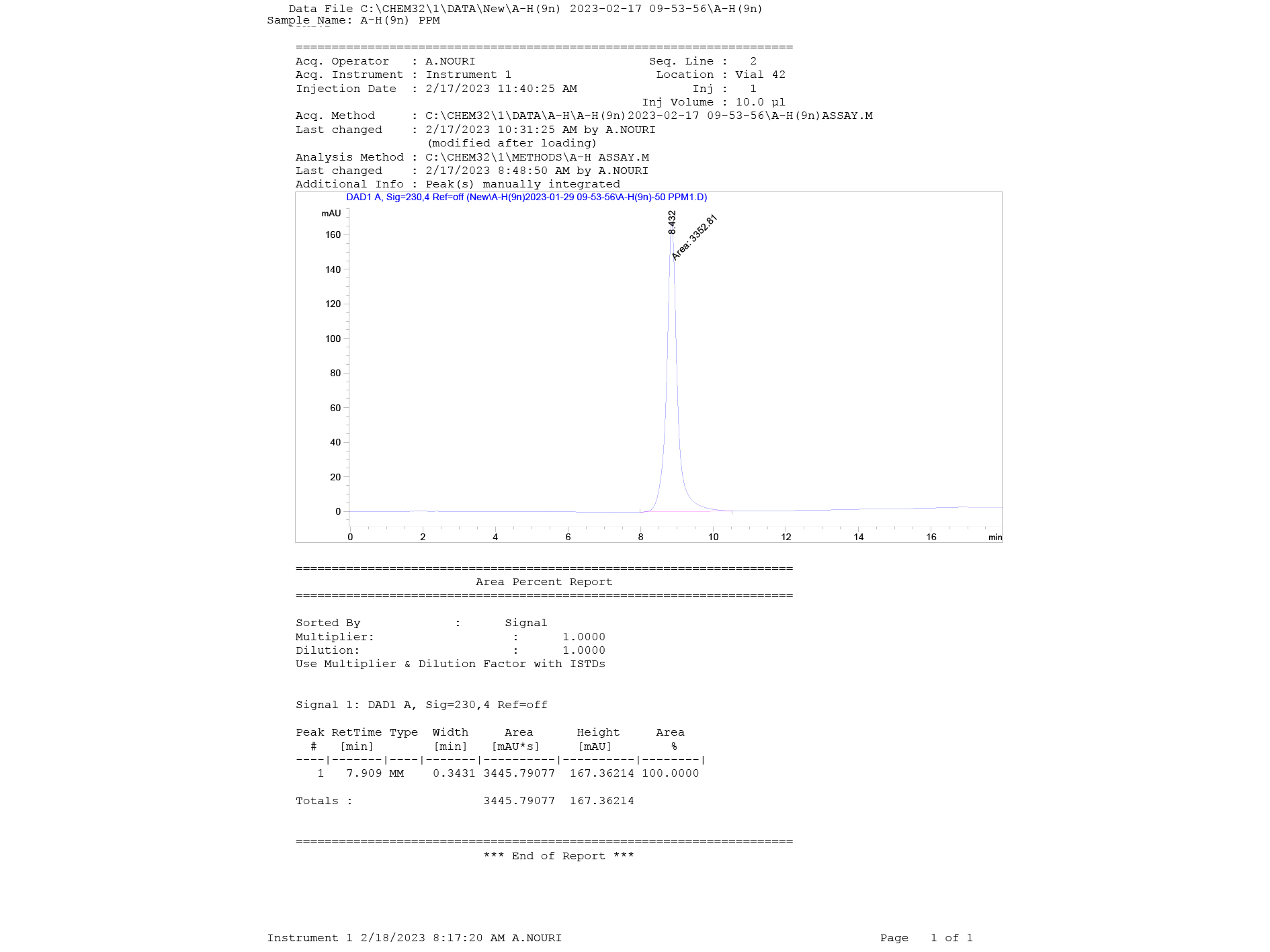


Figure. S3. HPLC results of 9p


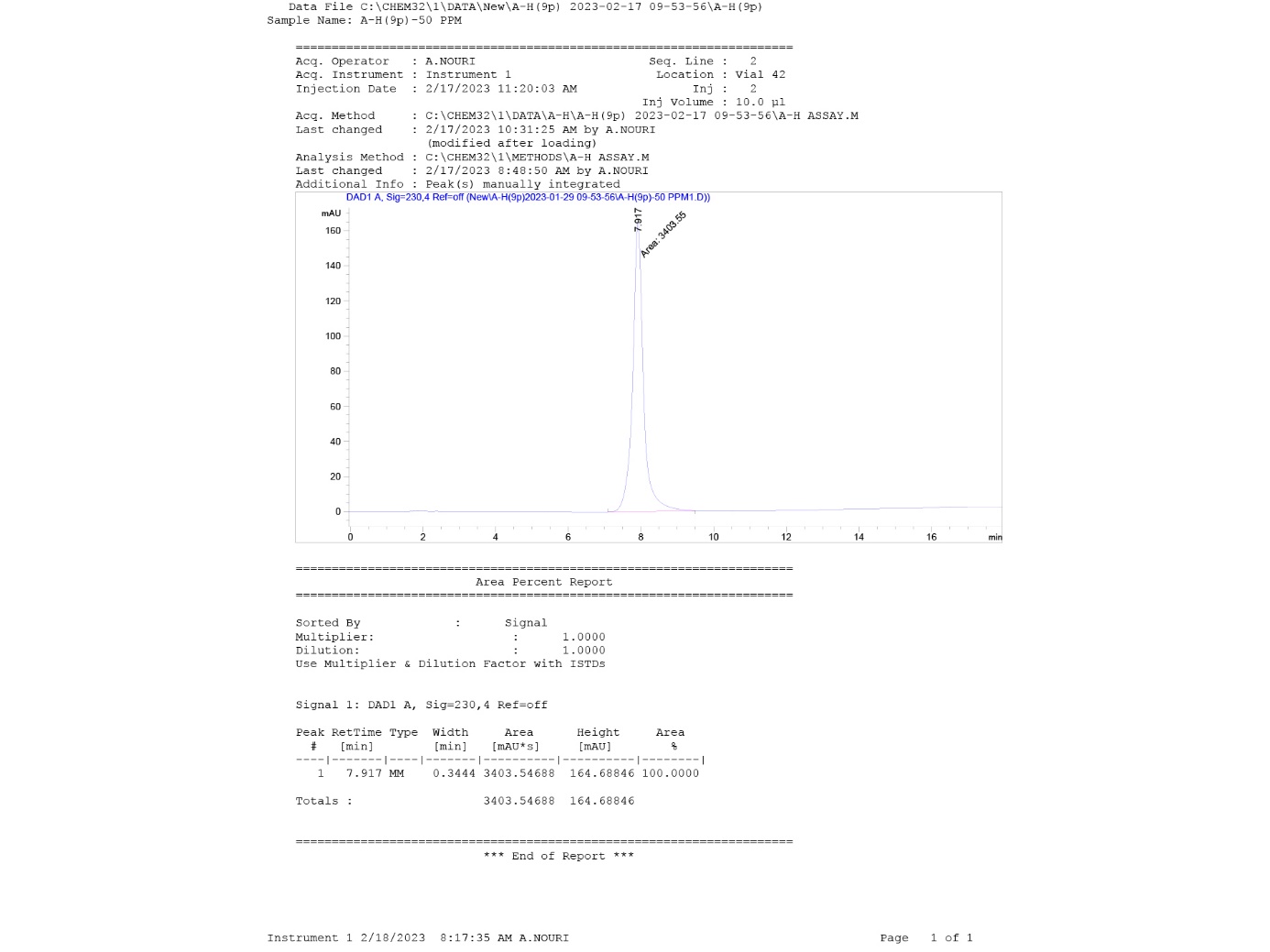


Figure S4. 1HNMR spectrum of compound 9a


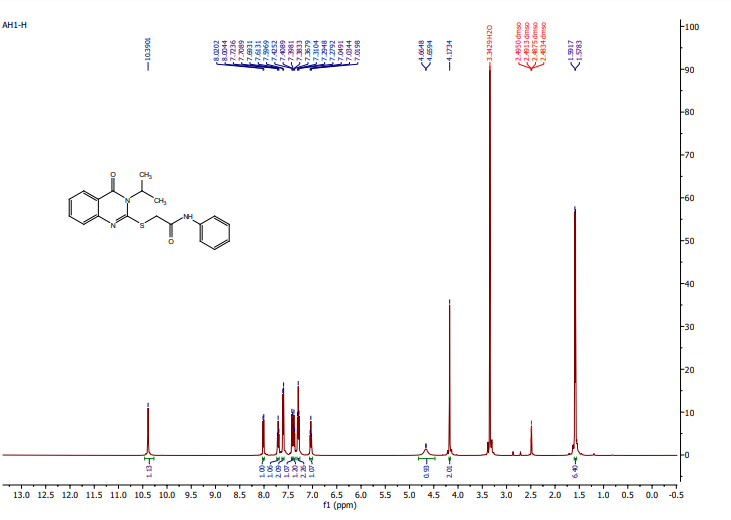


Figure S5. 13CNMR spectrum of compound 9a


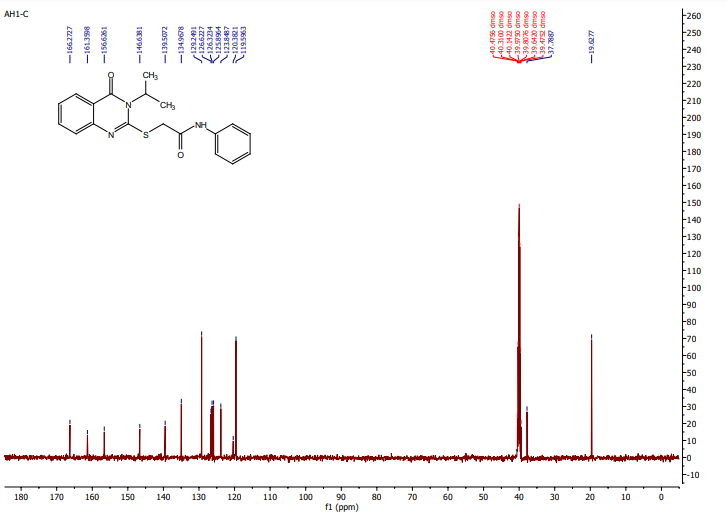


Figure S6. 1HNMR spectrum of compound 9b


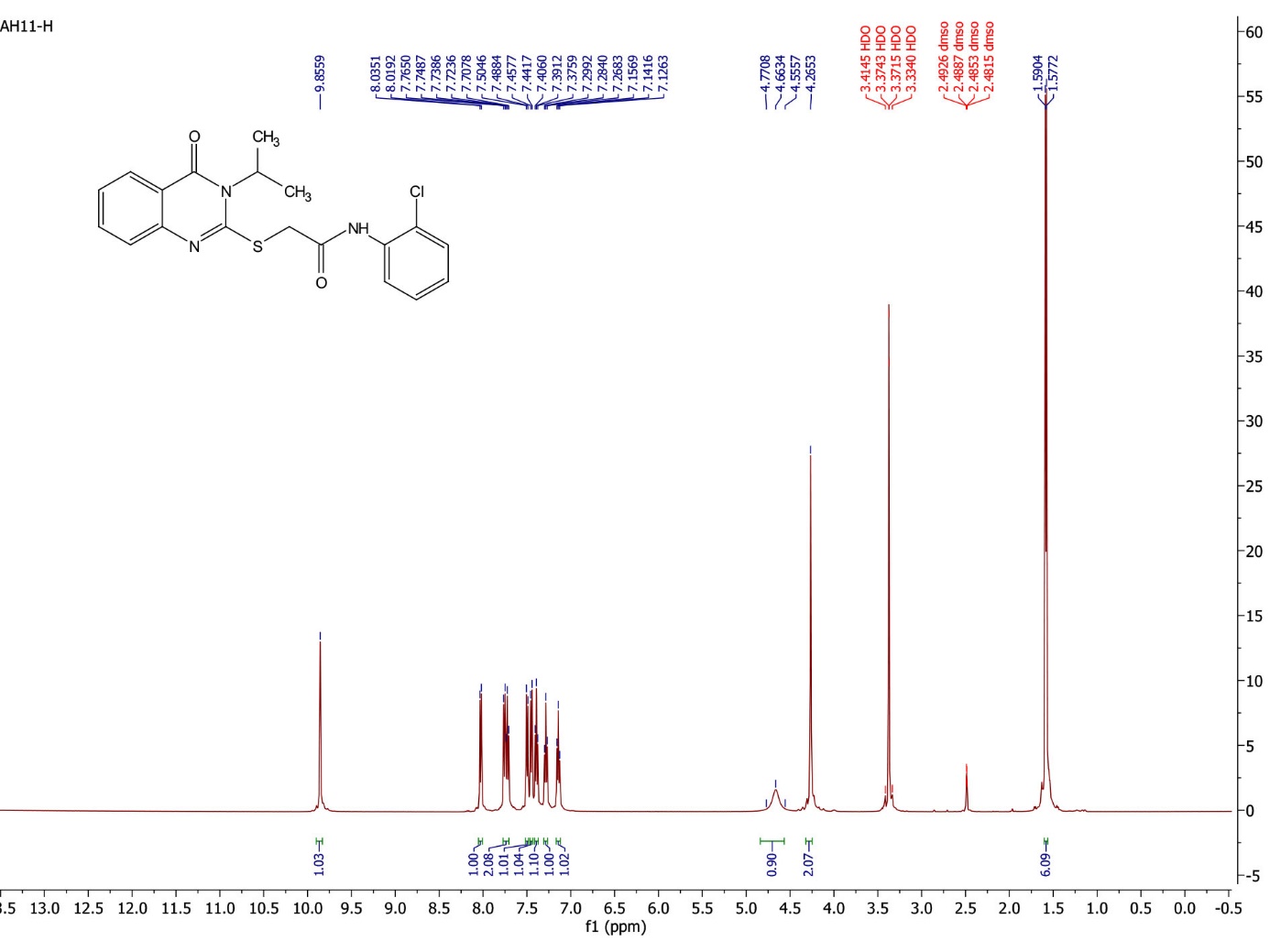


Figure S7. 13CNMR spectrum of compound 9b


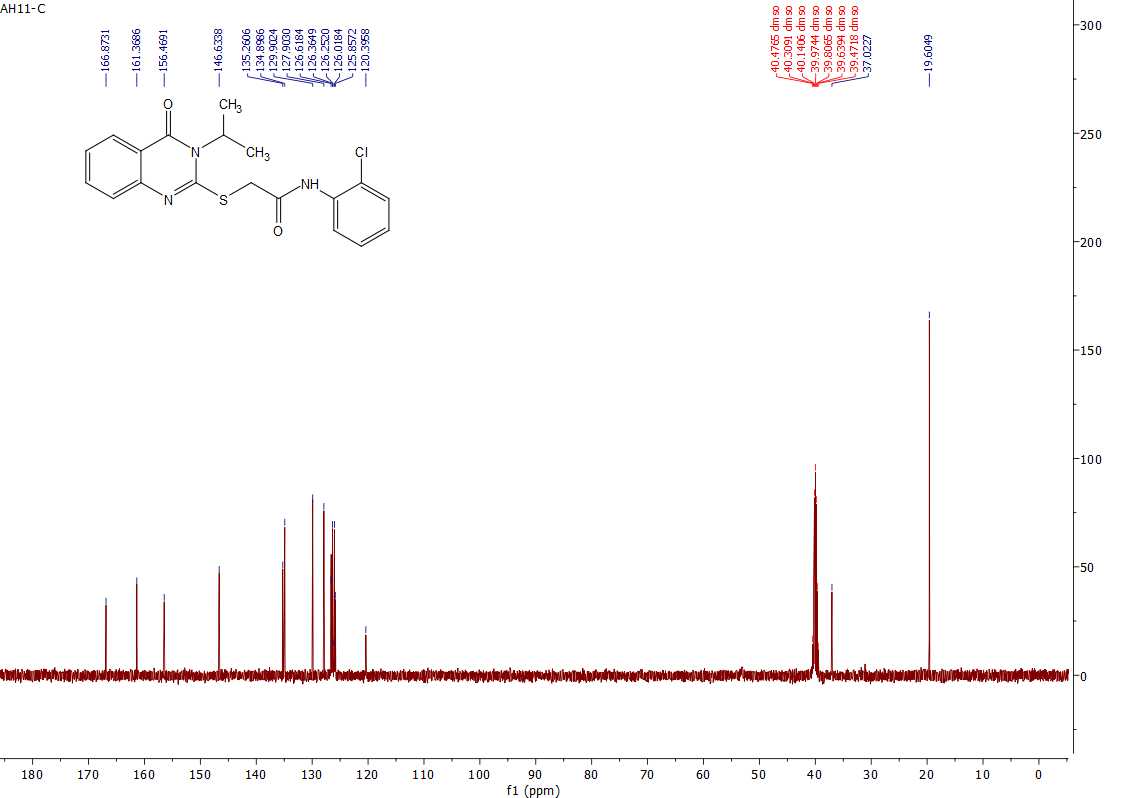


Figure S8. 1HNMR spectrum of compound 9c


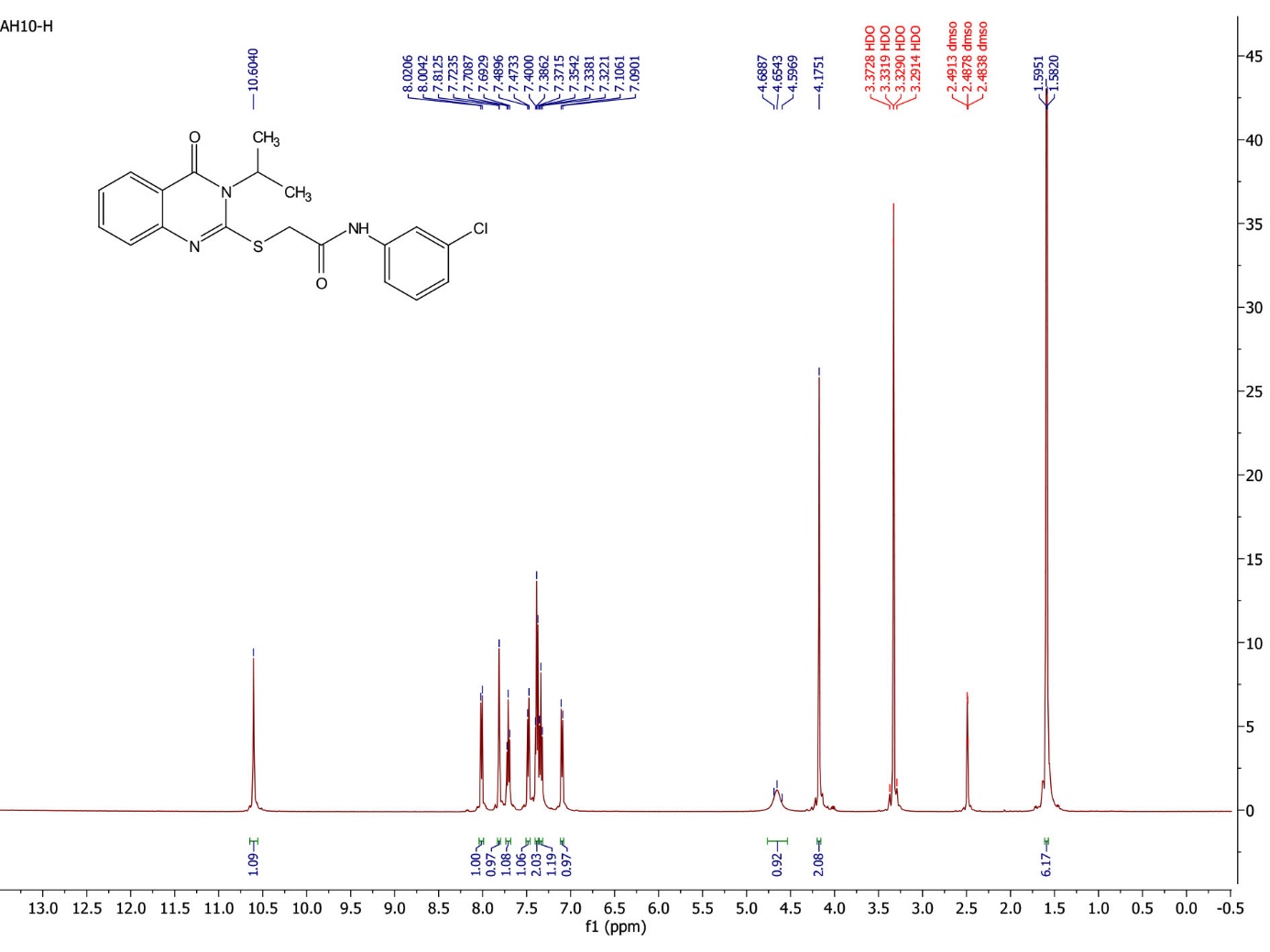


Figure S9. 13CNMR spectrum of compound 9c


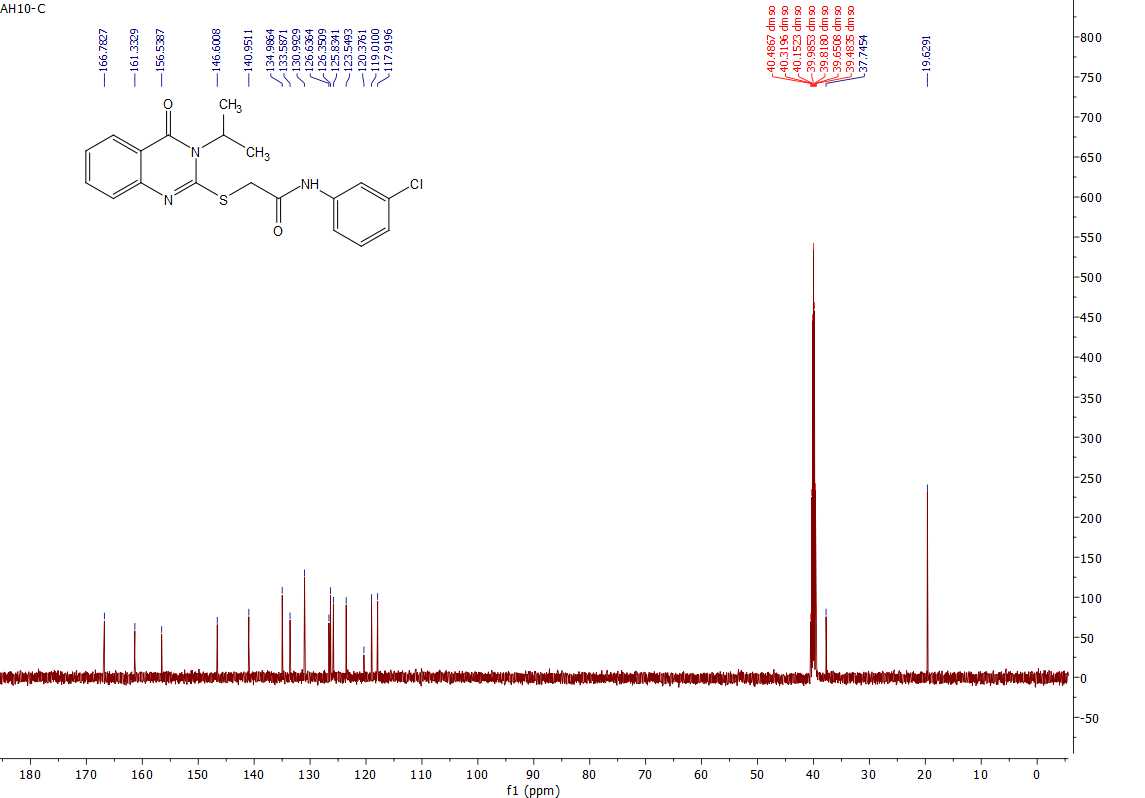


Figure S10. 1HNMR spectrum of compound 9d


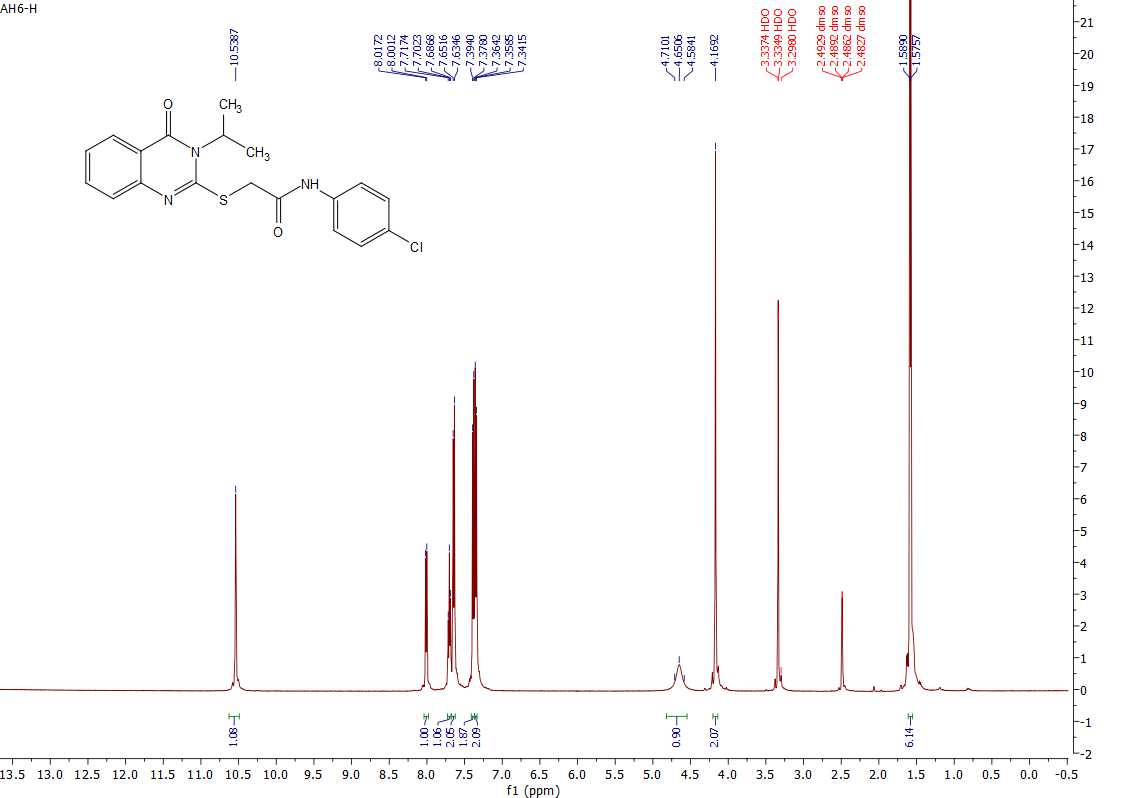


Figure S11. 13CNMR spectrum of compound 9d


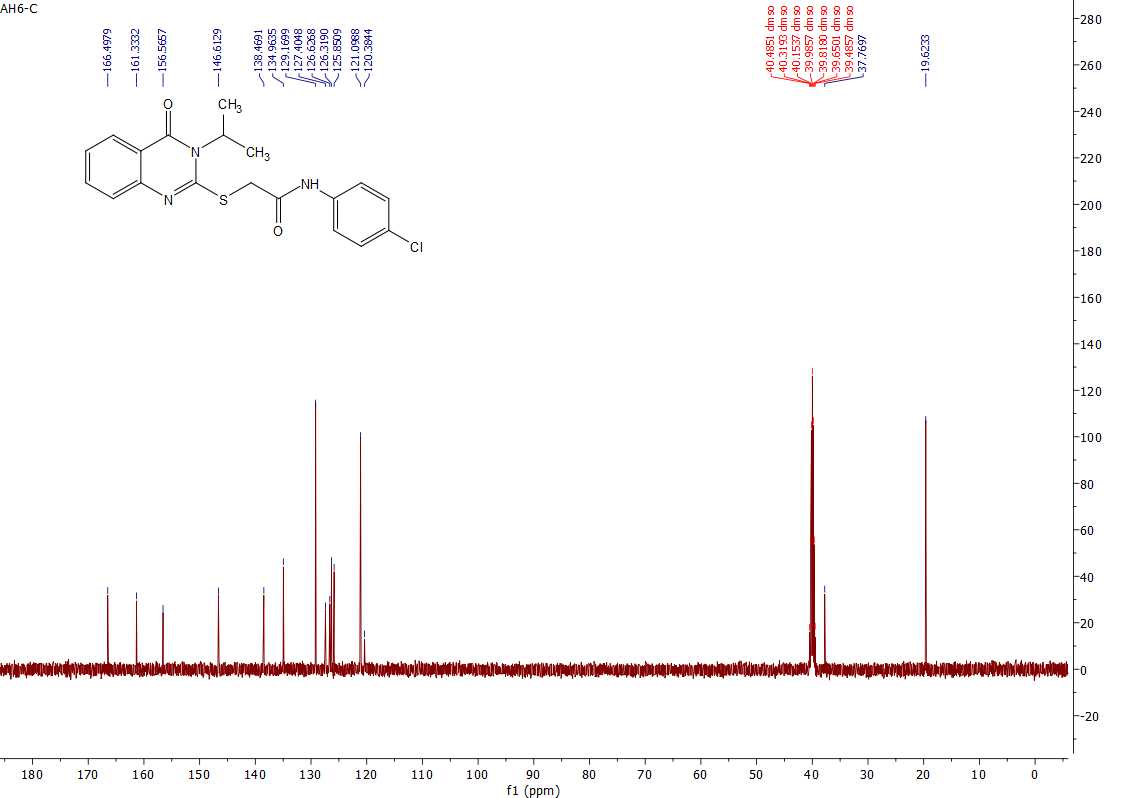


Figure S12. 1HNMR spectrum of compound 9e


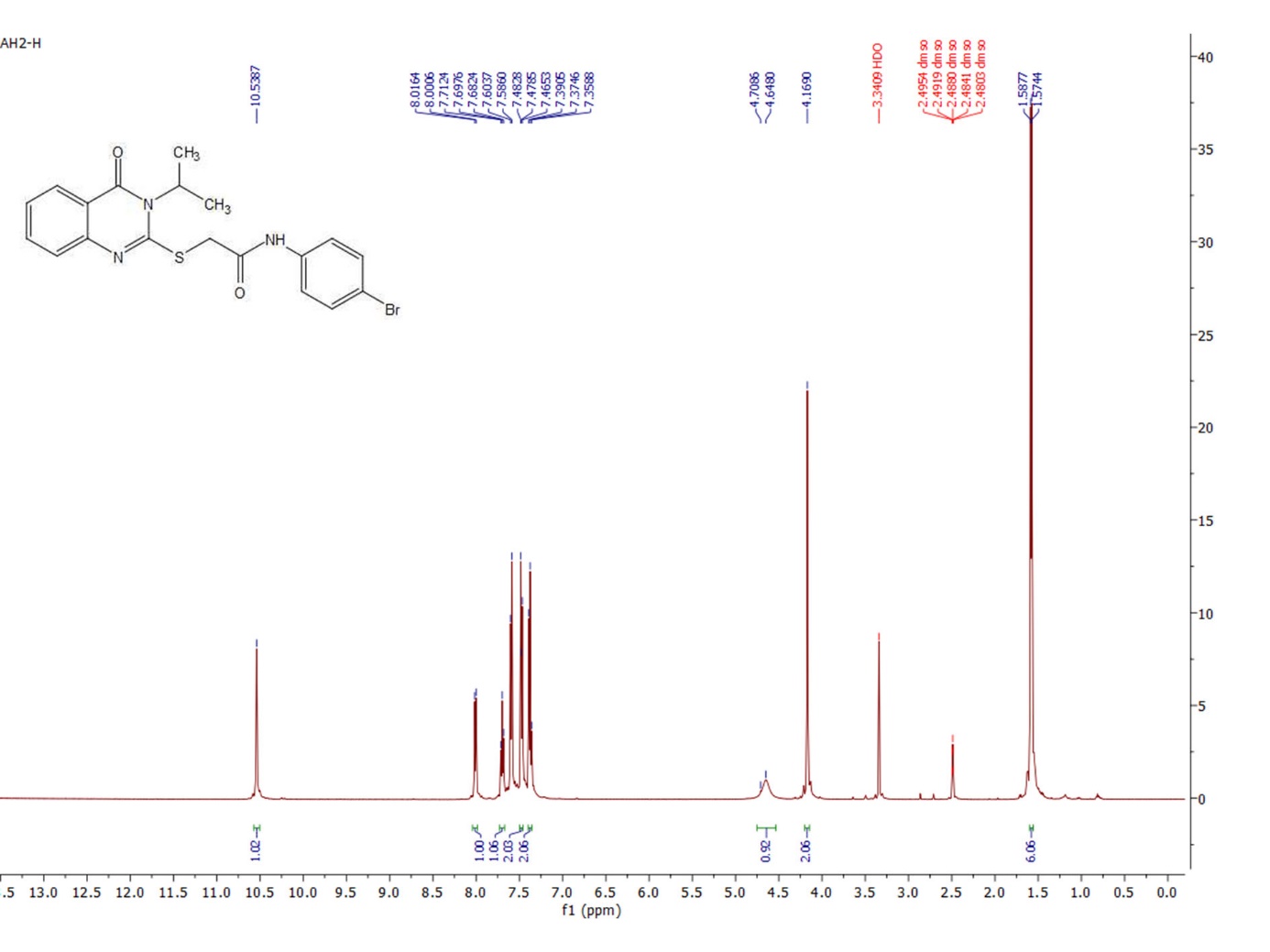


Figure S13. 13CNMR spectrum of compound 9e


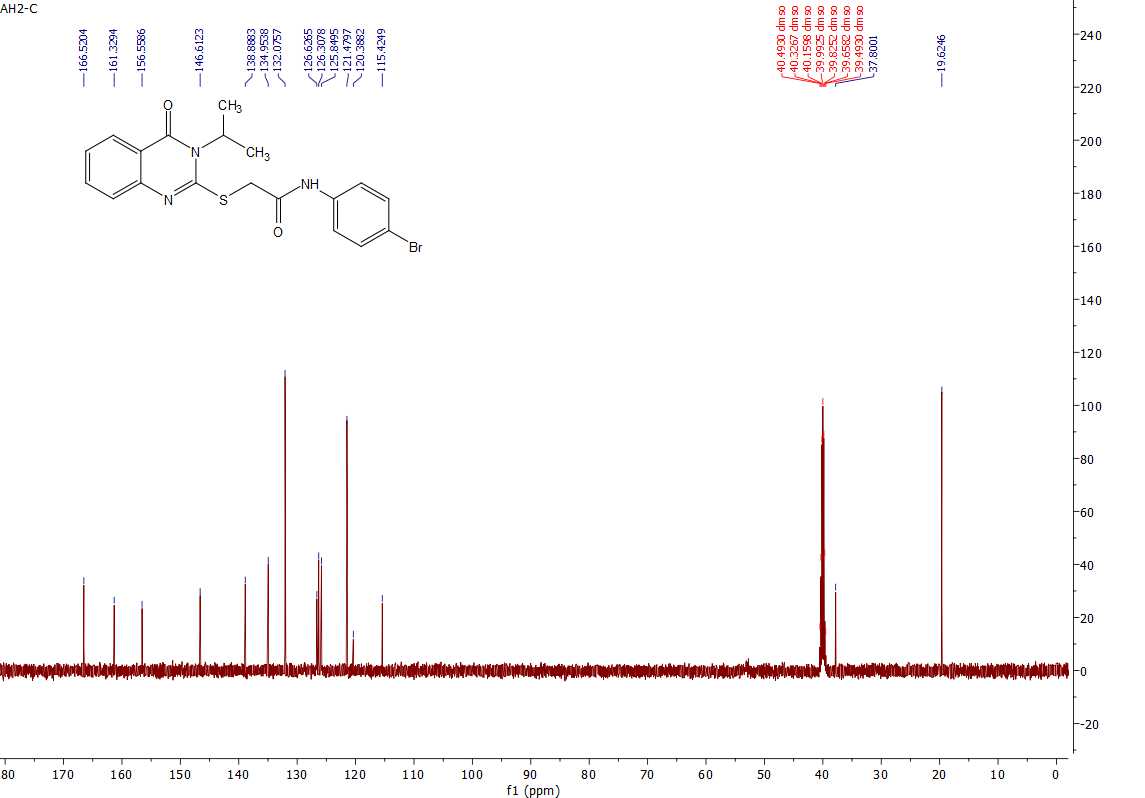


Figure S14. 1HNMR spectrum of compound 9f


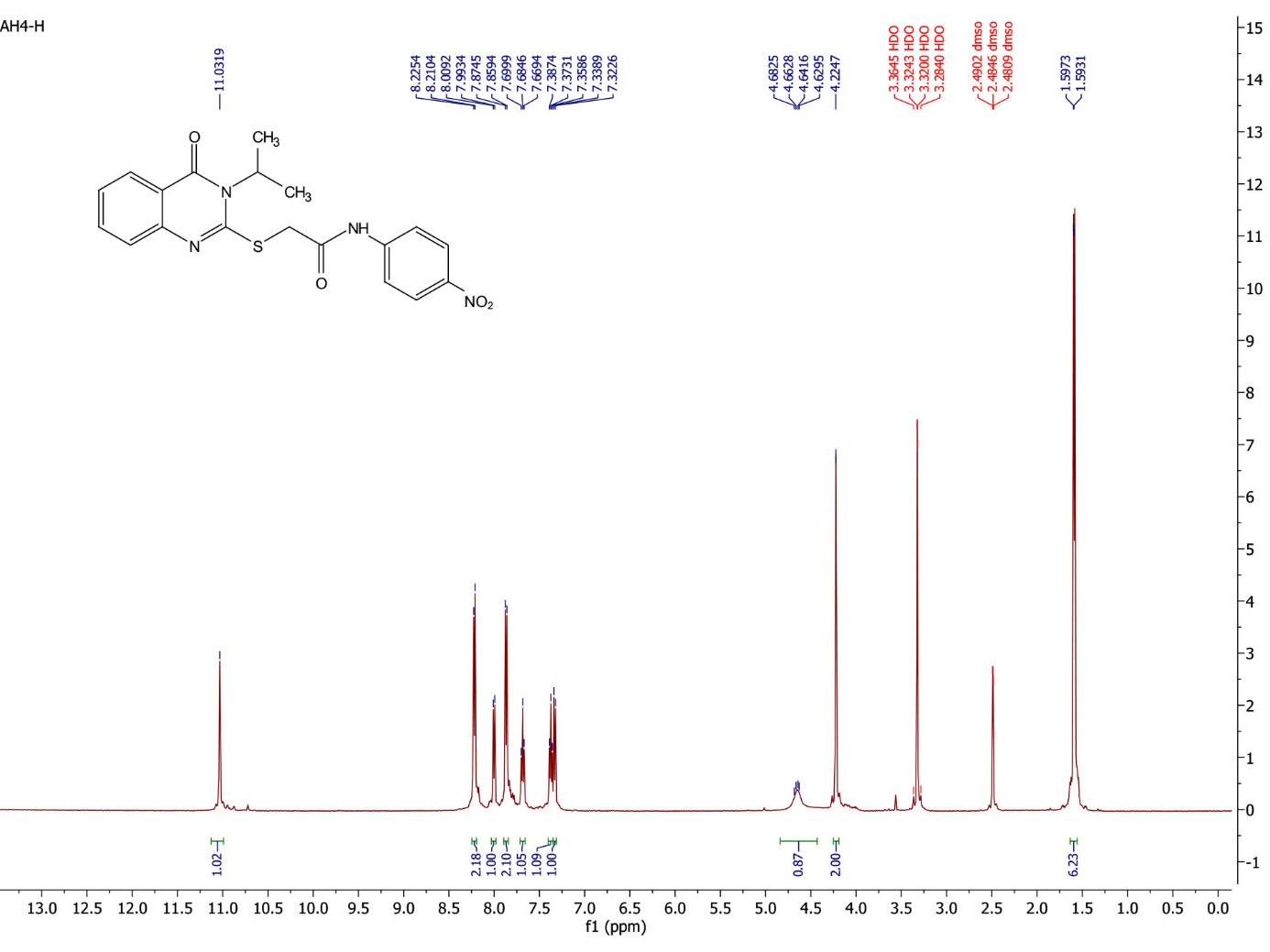


Figure S15. 13CNMR spectrum of compound 9f


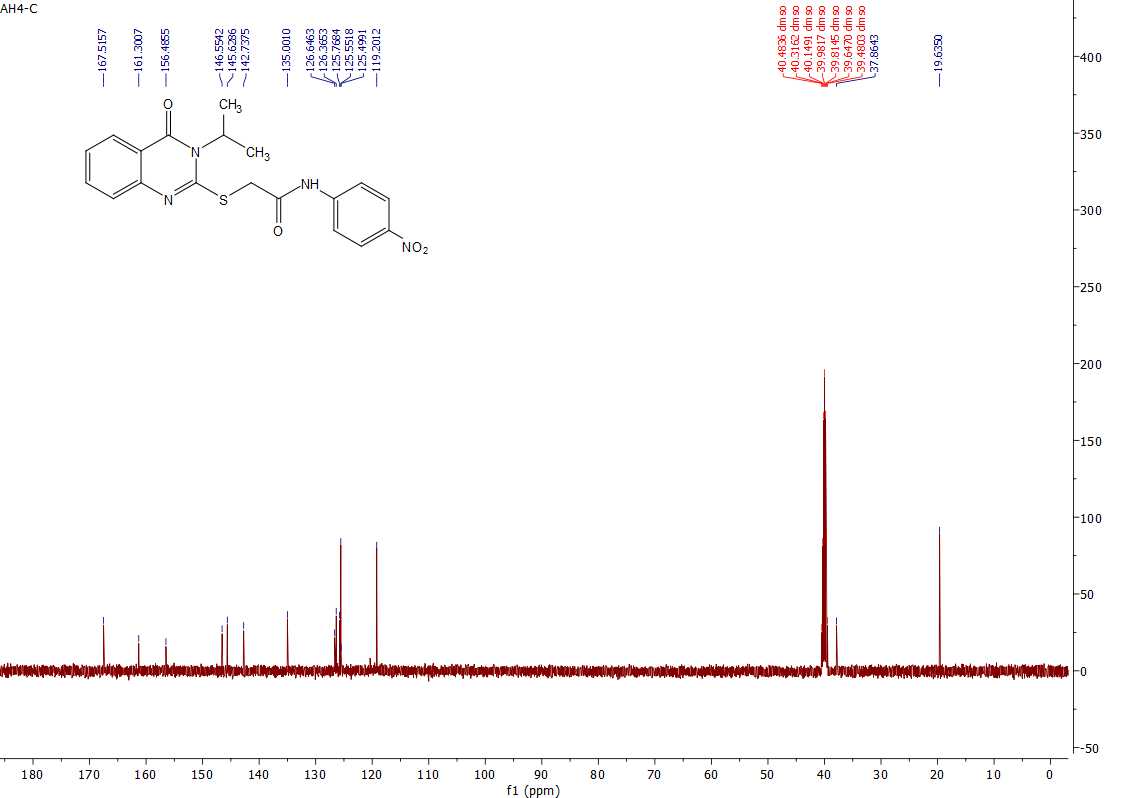


Figure S16. 1HNMR spectrum of compound 9g


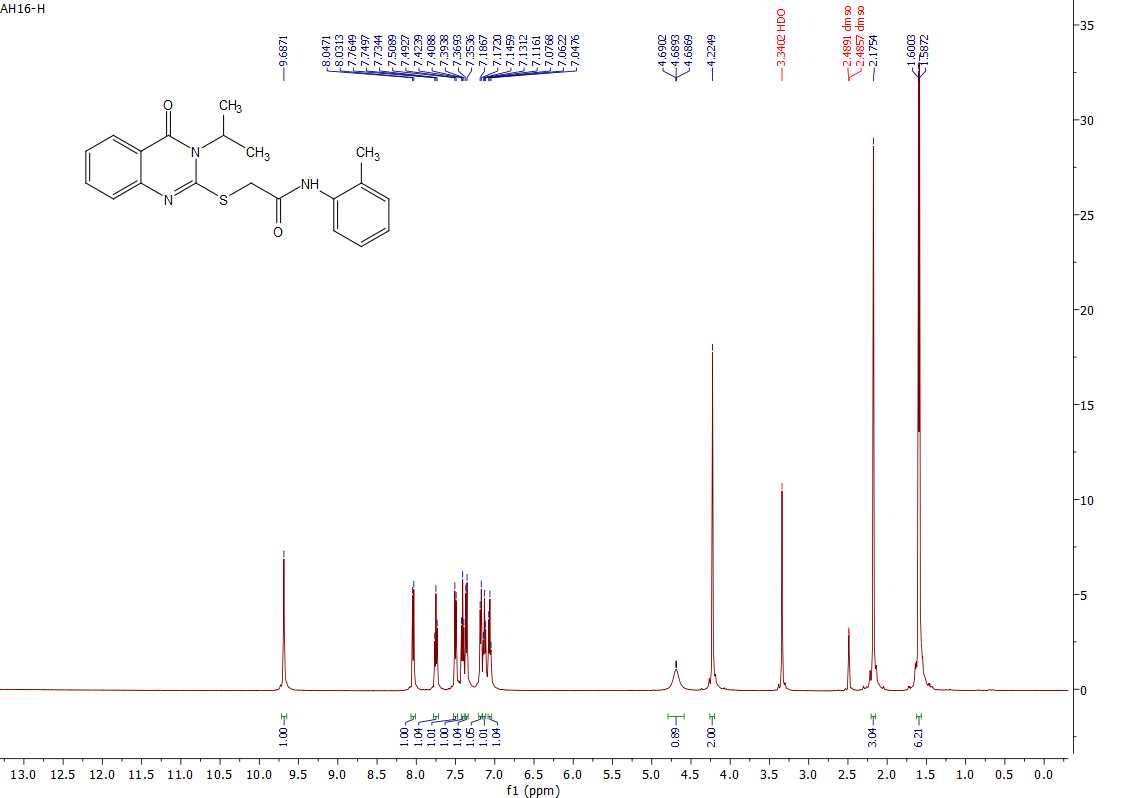


Figure S17. 13CNMR spectrum of compound 9g


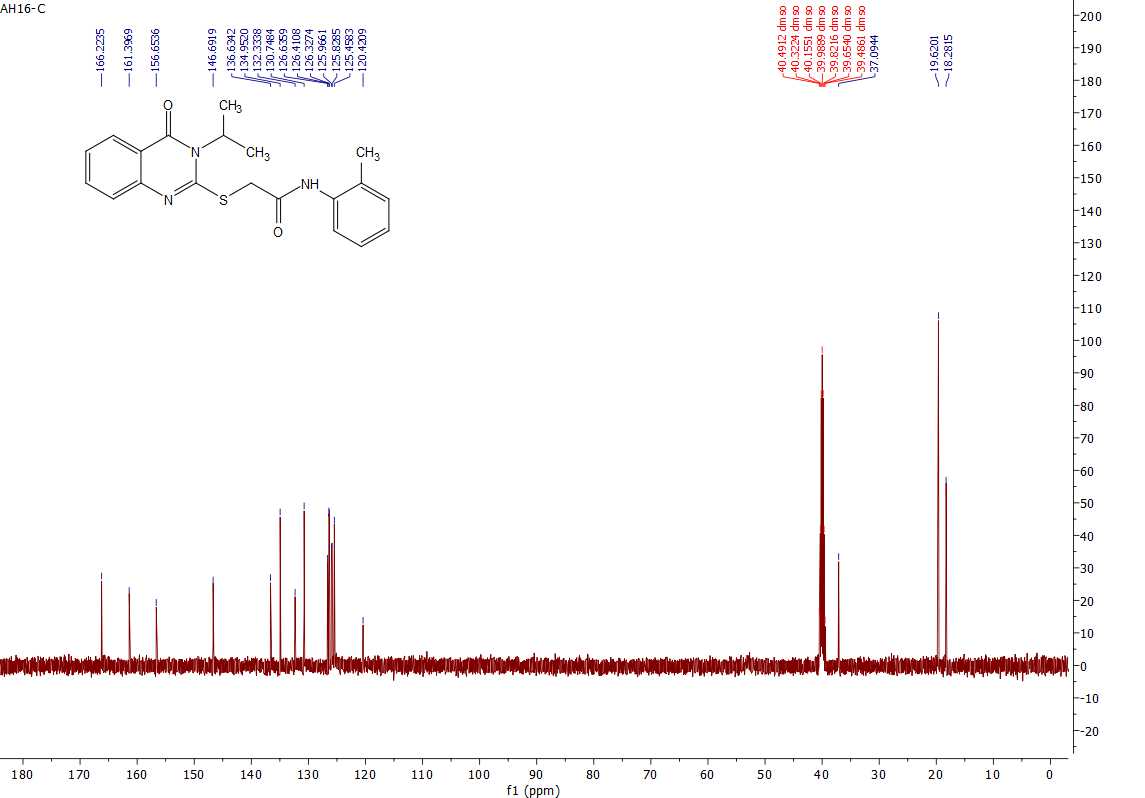


Figure S18. 1HNMR spectrum of compound 9h


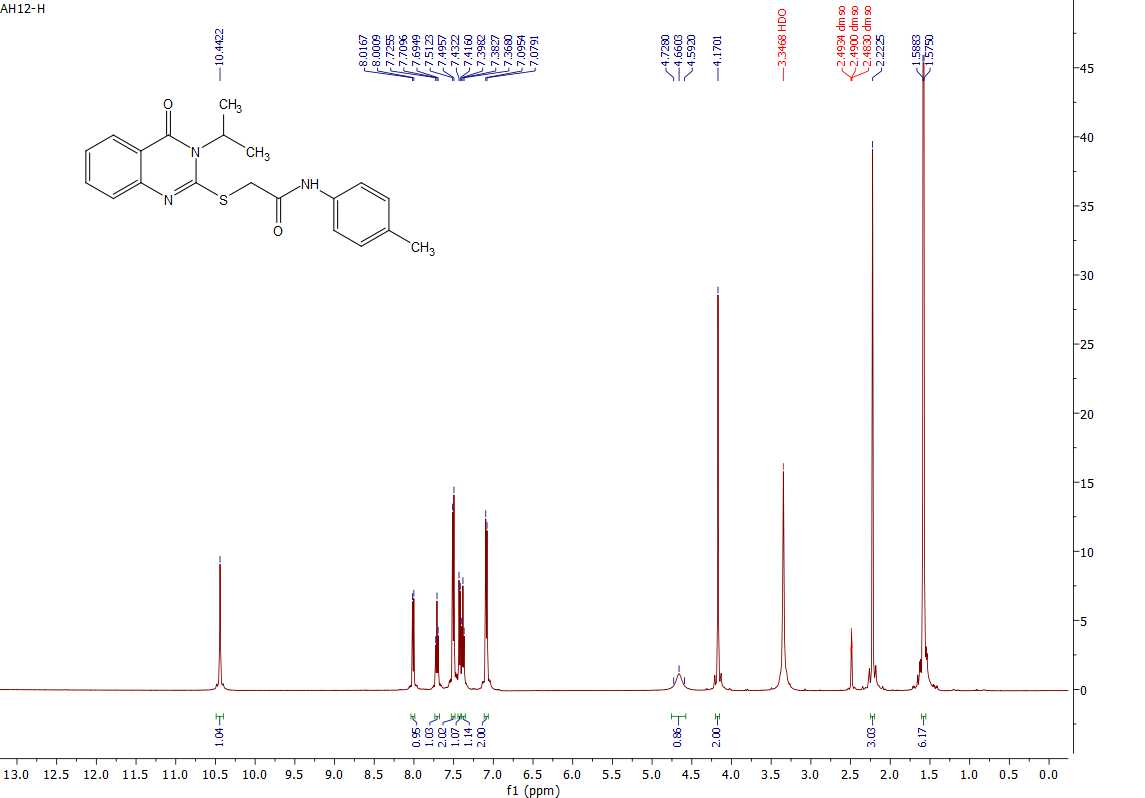


Figure S19. 13CNMR spectrum of compound 9h


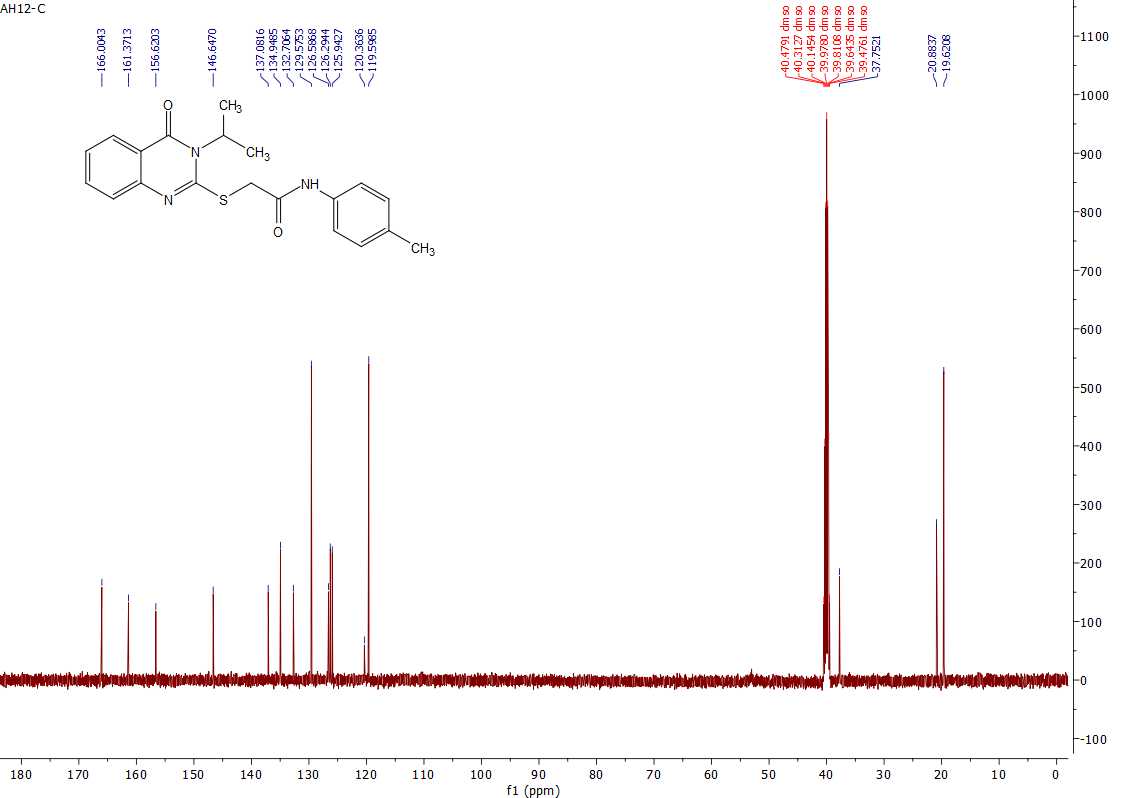


Figure S20. 1HNMR spectrum of compound 9i


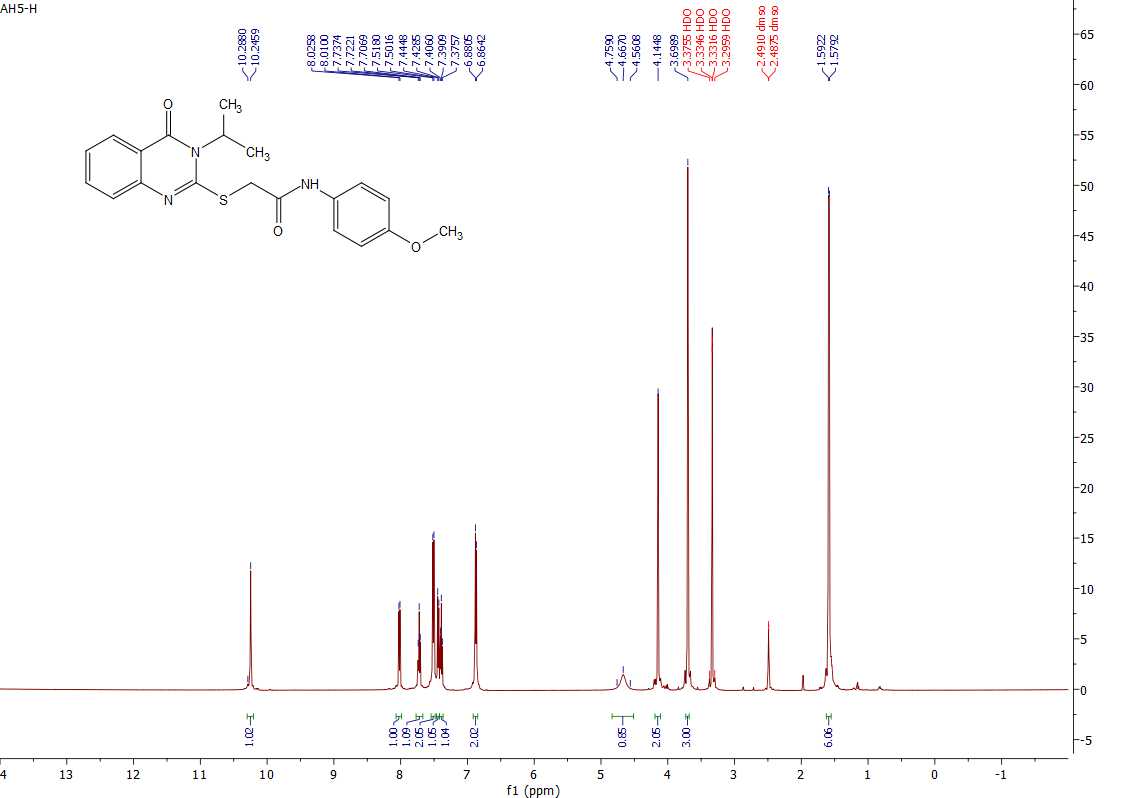


Figure S21. 13CNMR spectrum of compound 9i


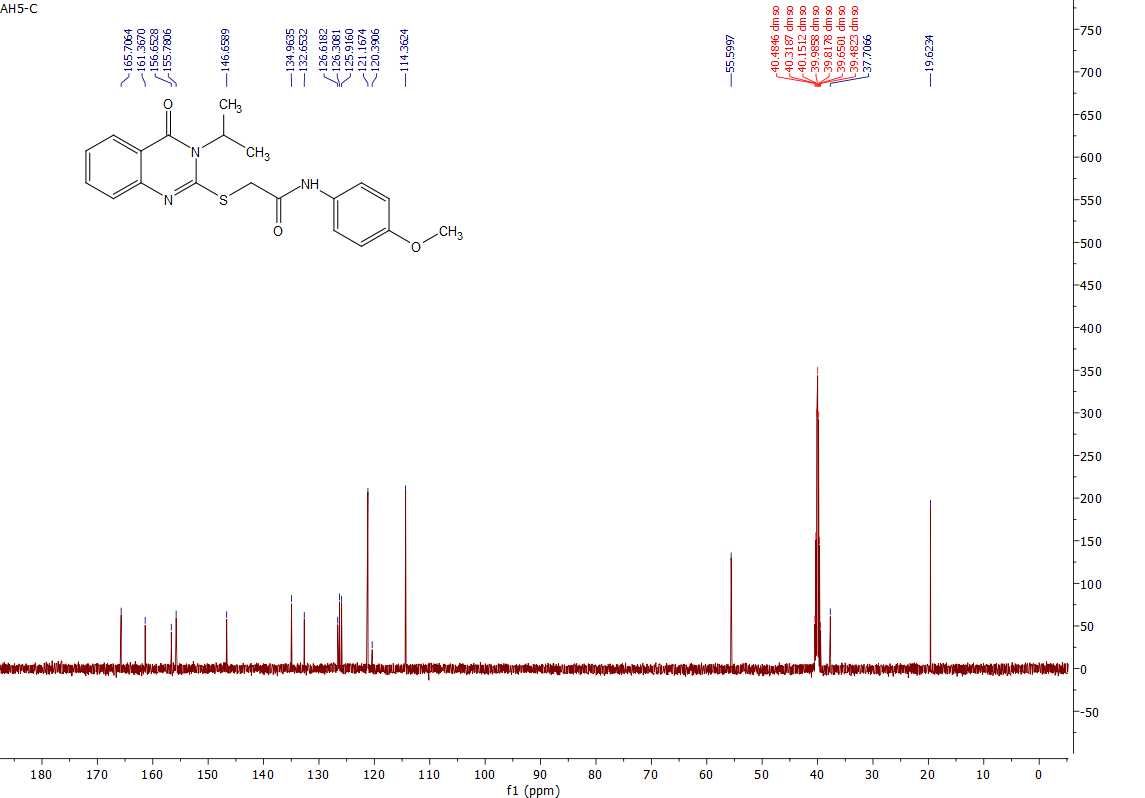


Figure S22. 1HNMR spectrum of compound 9j


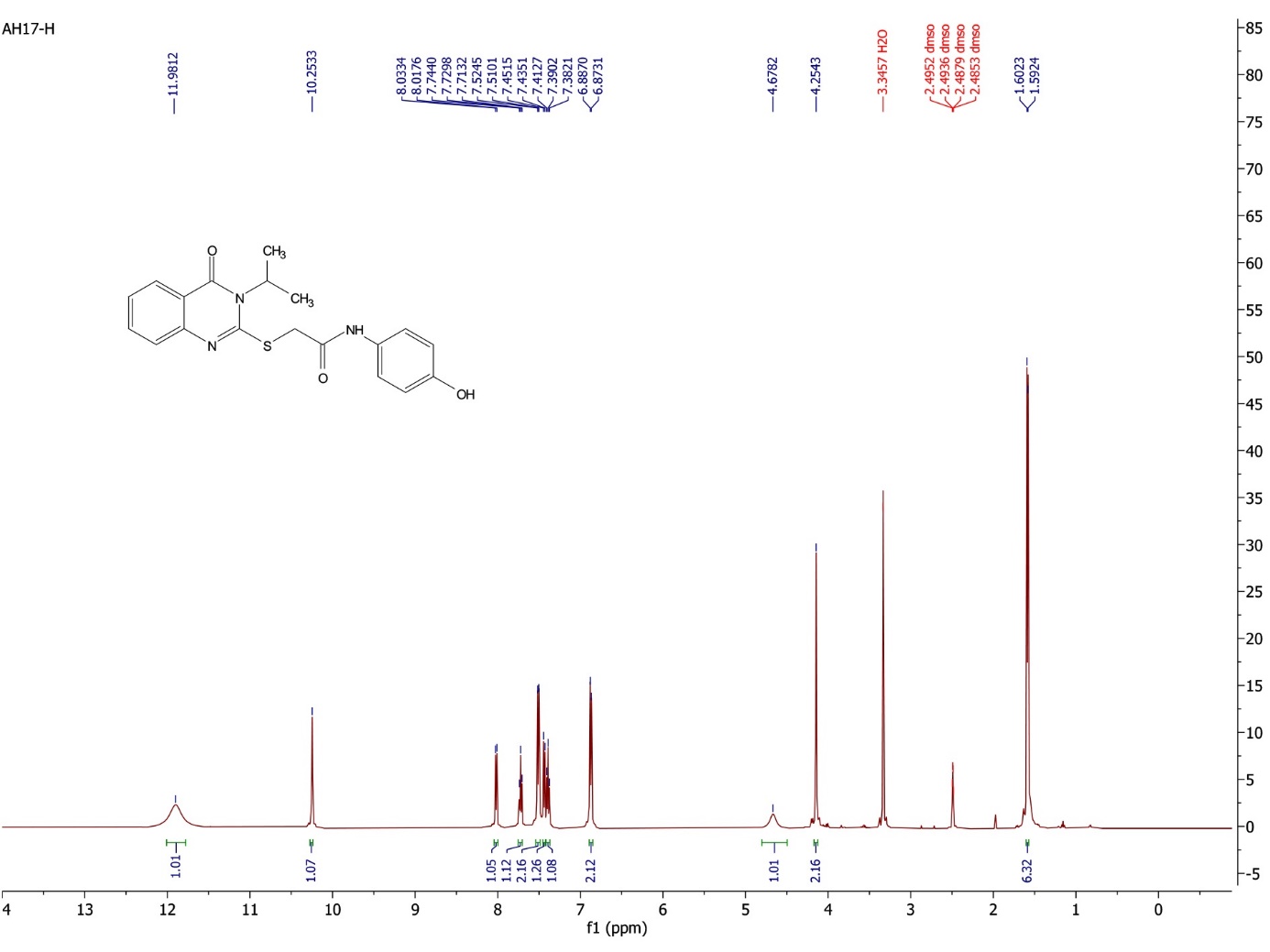


Figure S23. 13CNMR spectrum of compound 9j


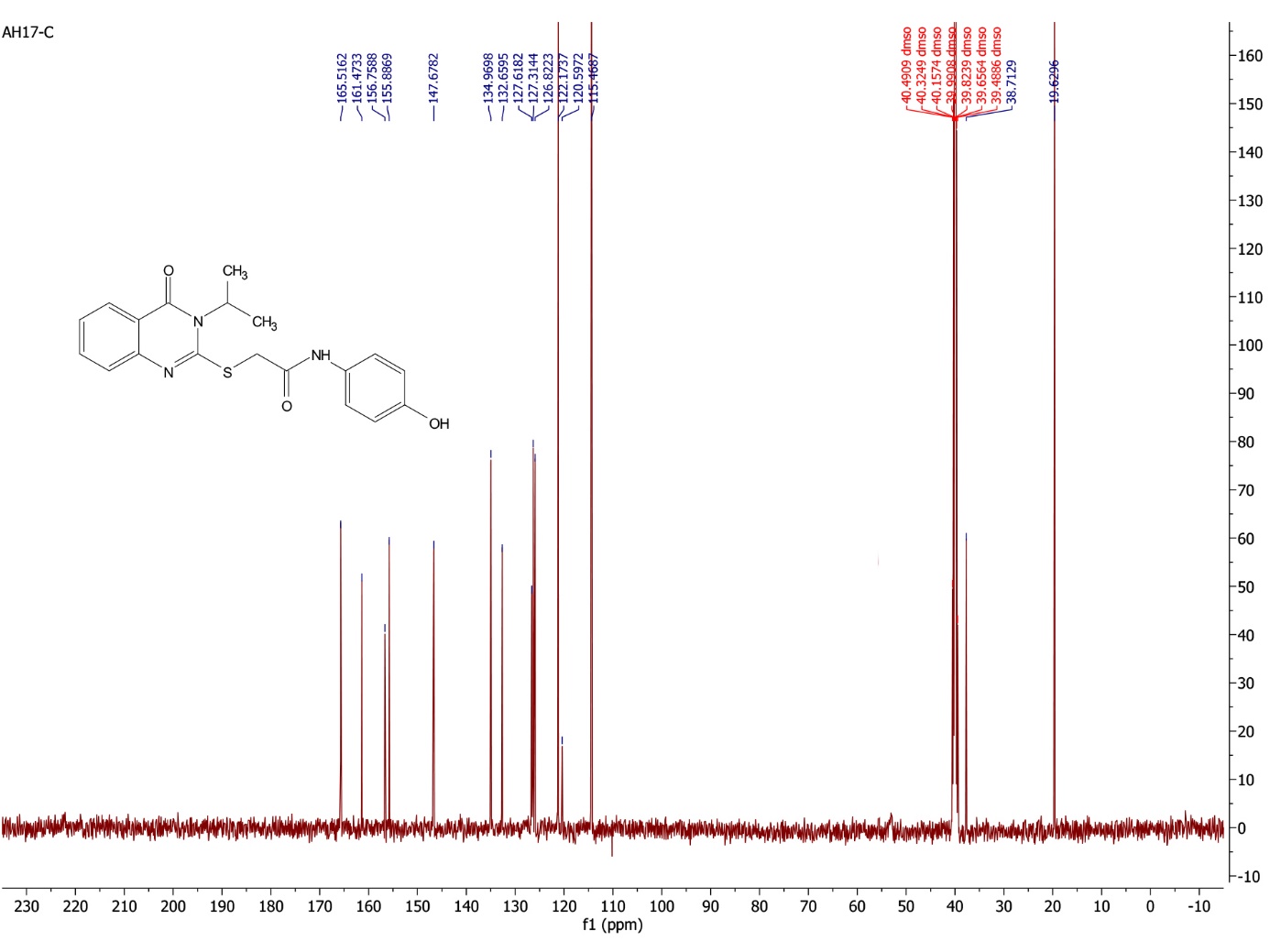


Figure S24. 1HNMR spectrum of compound 9k


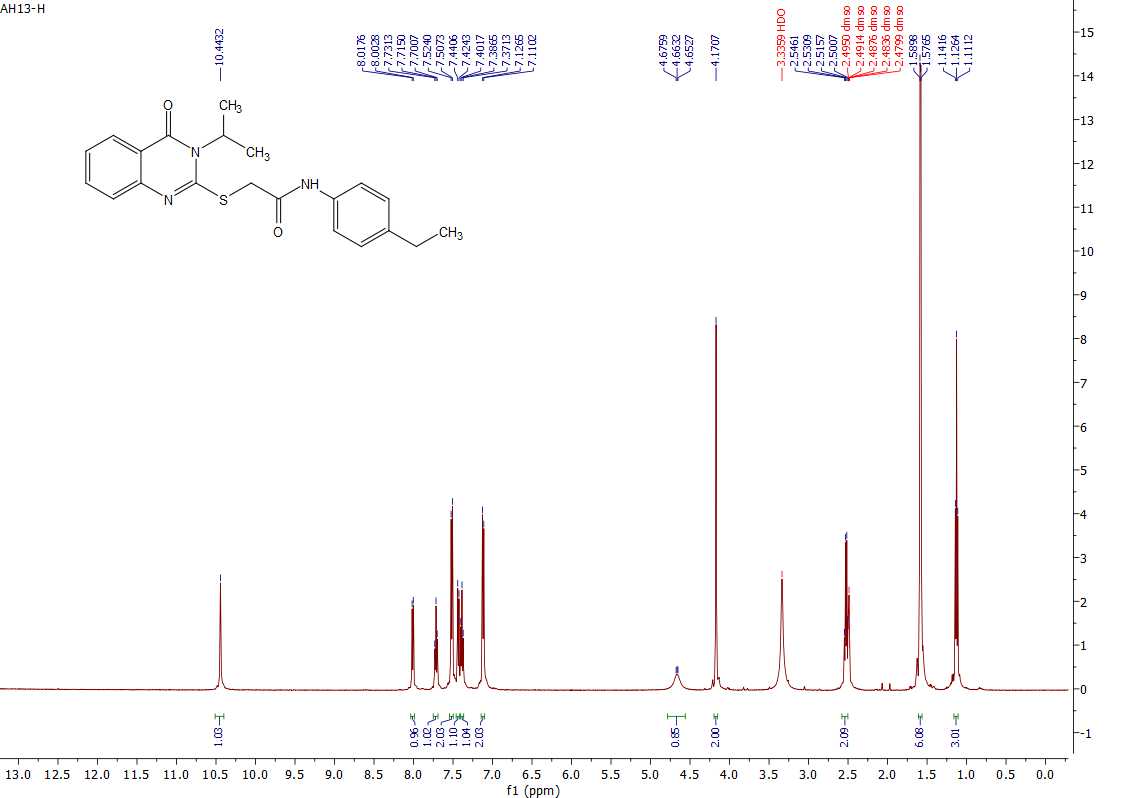


Figure S25. 13CNMR spectrum of compound 9k


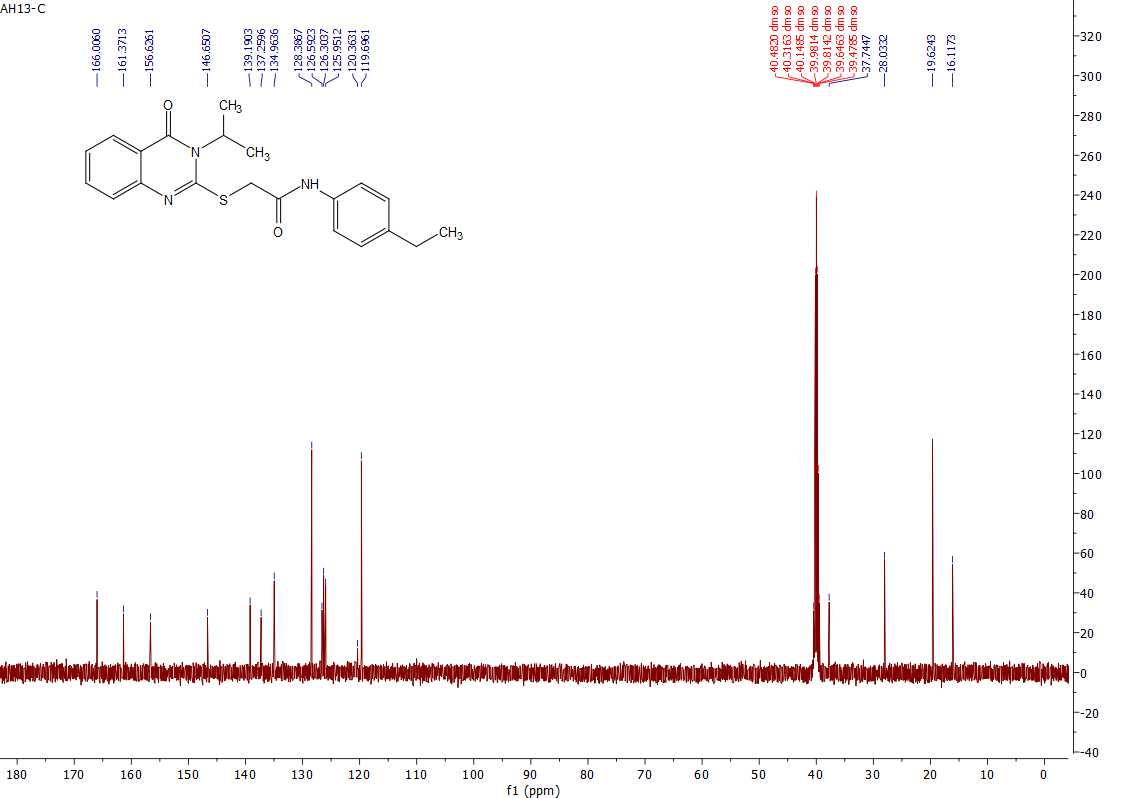


Figure S26. 1HNMR spectrum of compound 9l


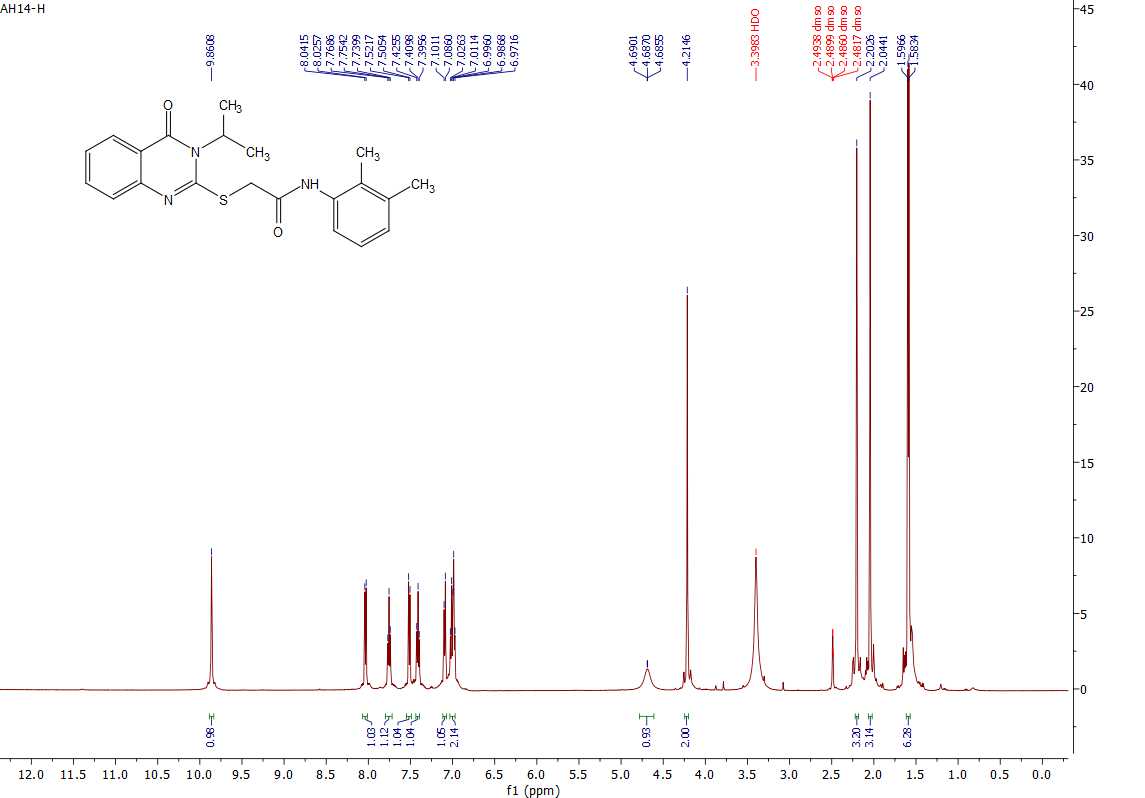


Figure S27. ^13^CNMR spectrum of compound 9l


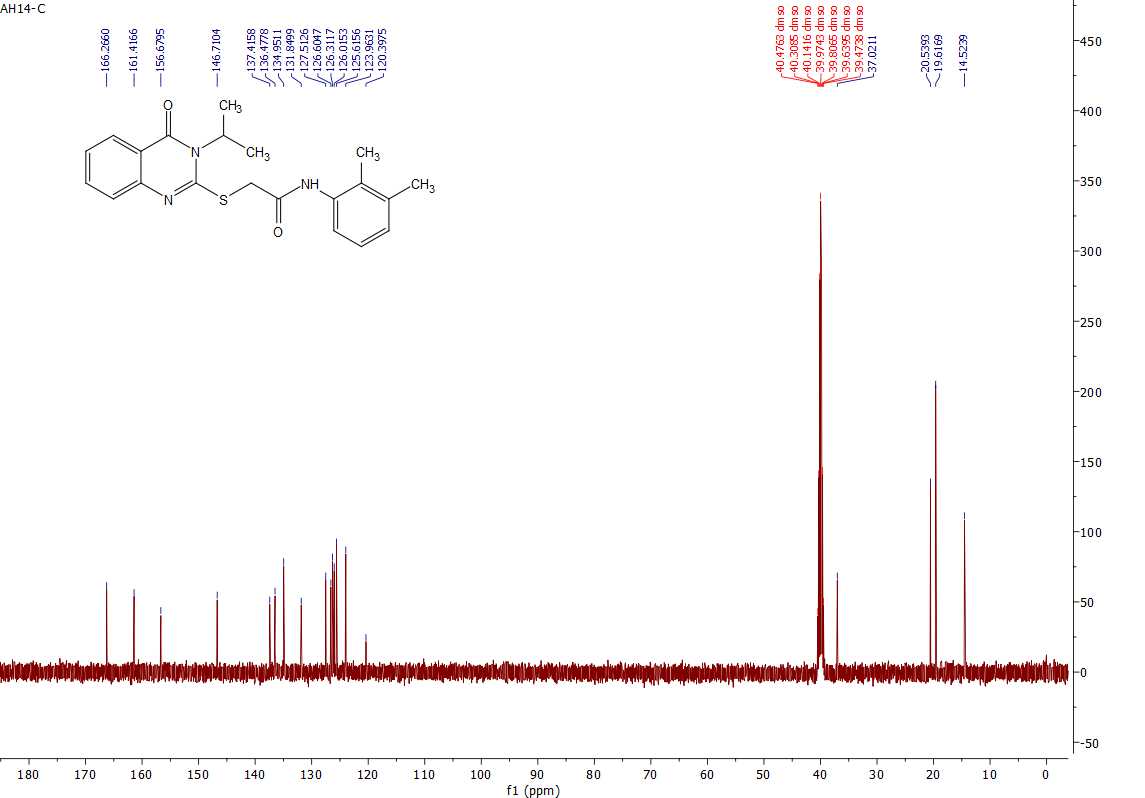


Figure S28. 1HNMR spectrum of compound 9m


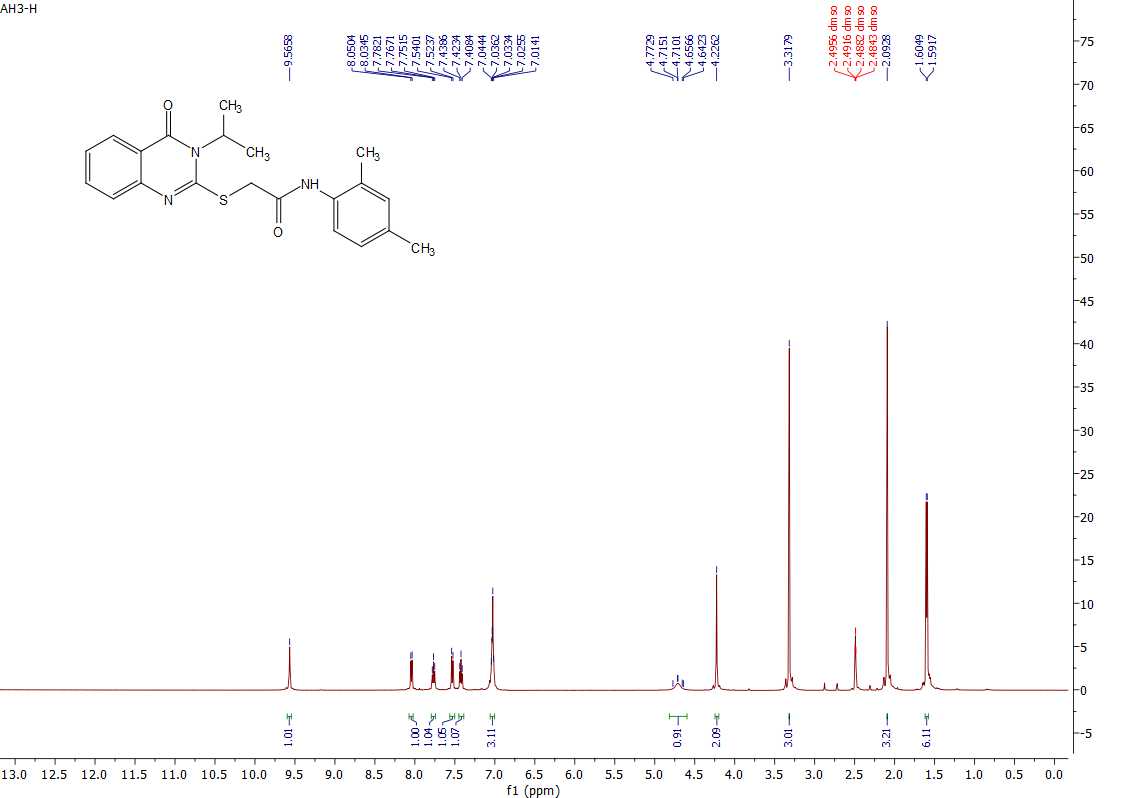


Figure S29. 13CNMR spectrum of compound 9m


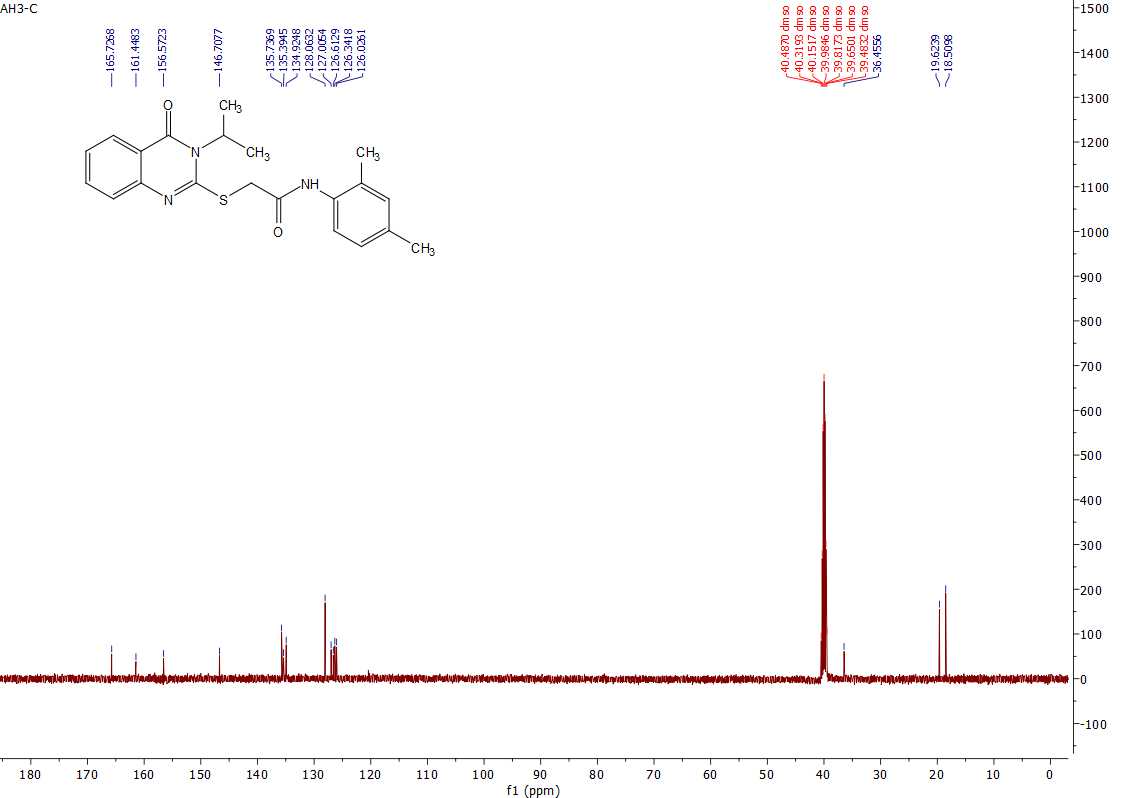


Figure S30. 1HNMR spectrum of compound 9n


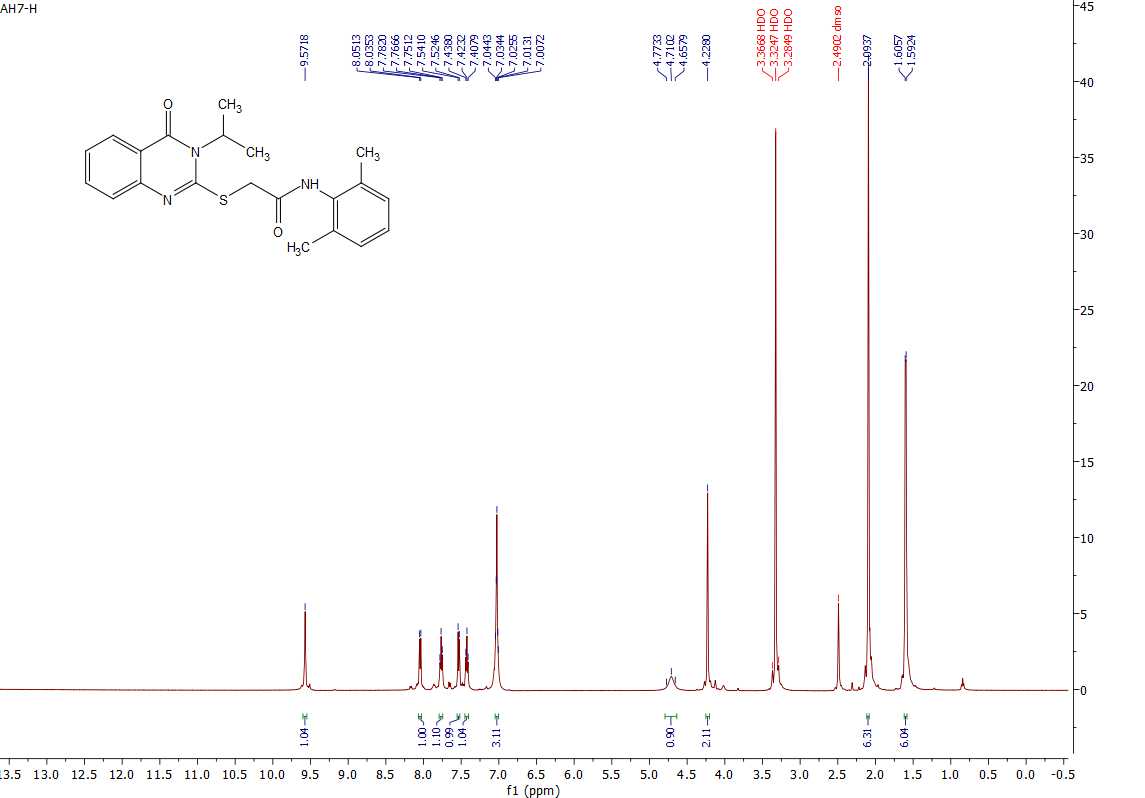


Figure S31. 13CNMR spectrum of compound 9n


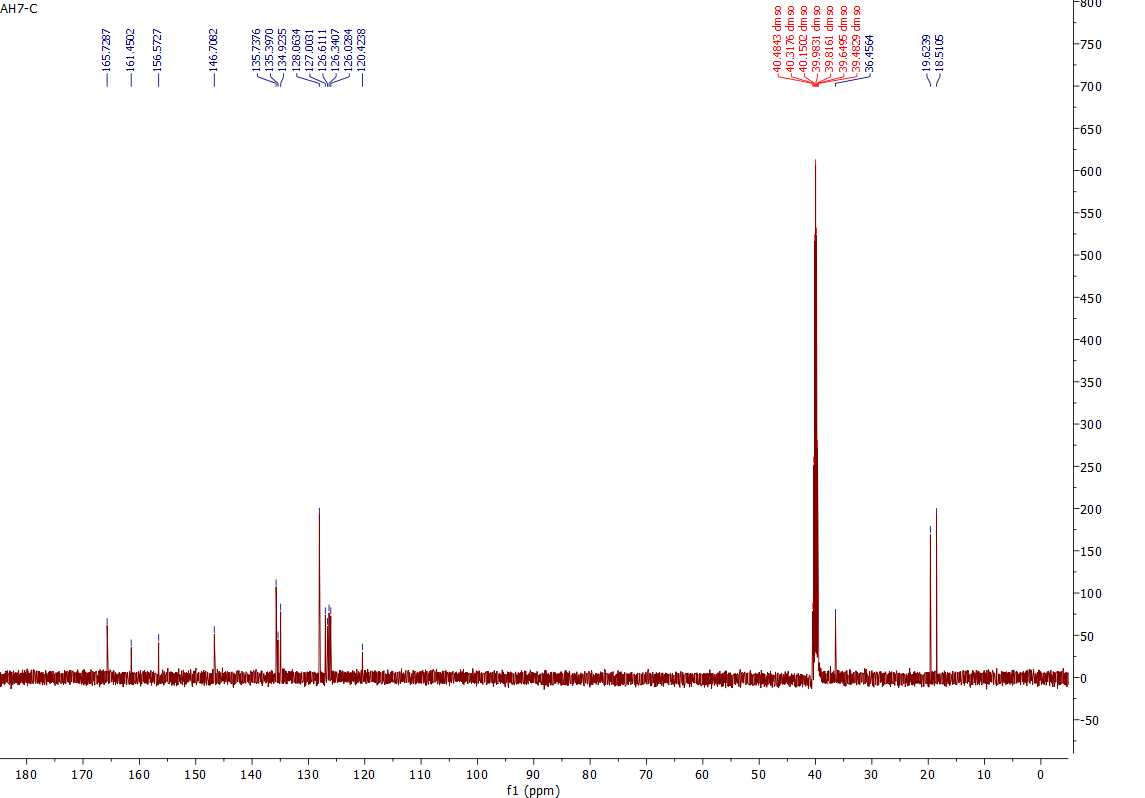


Figure S32. 1HNMR spectrum of compound 9o


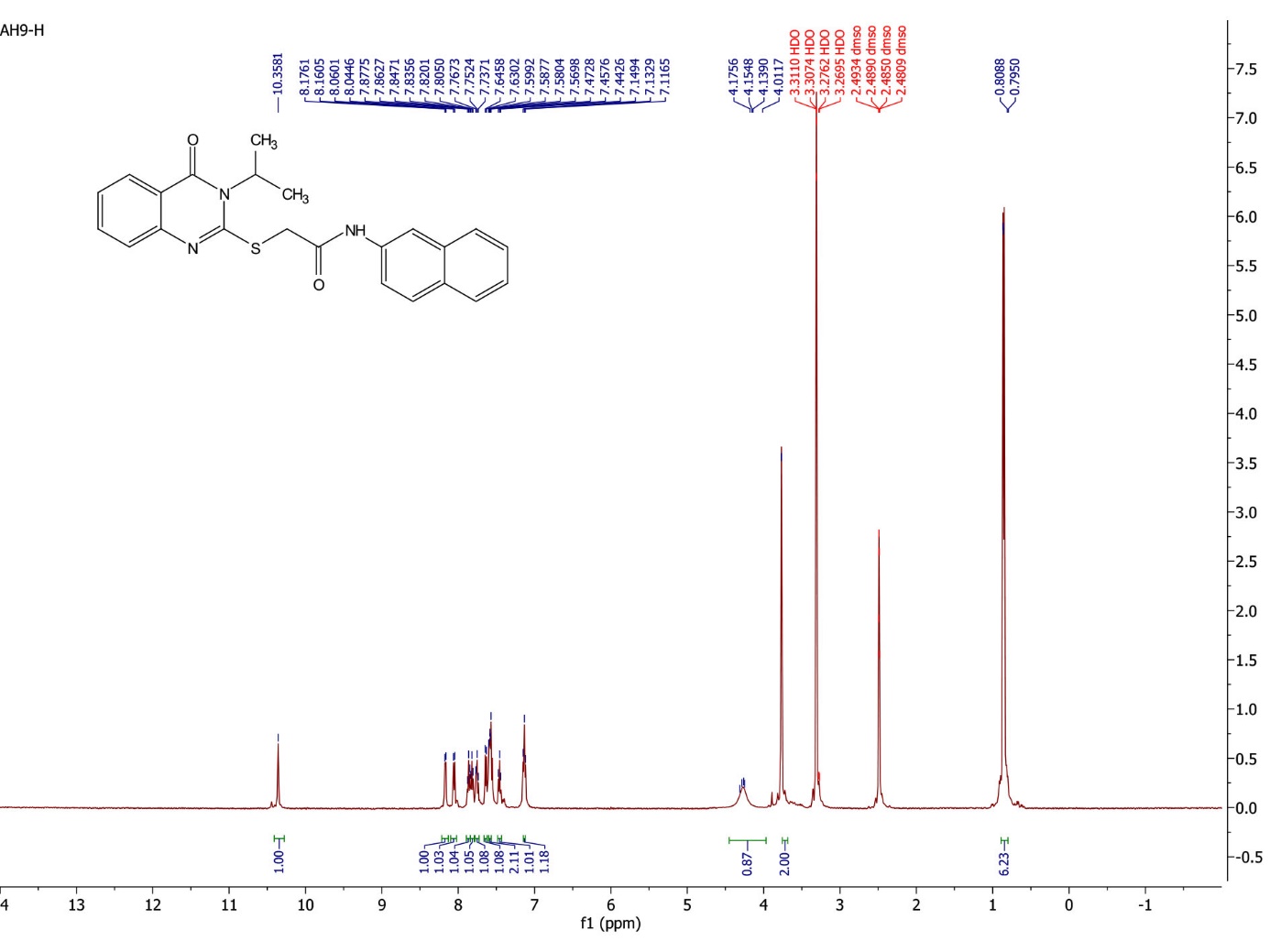


Figure S33. 13CNMR spectrum of compound 9o


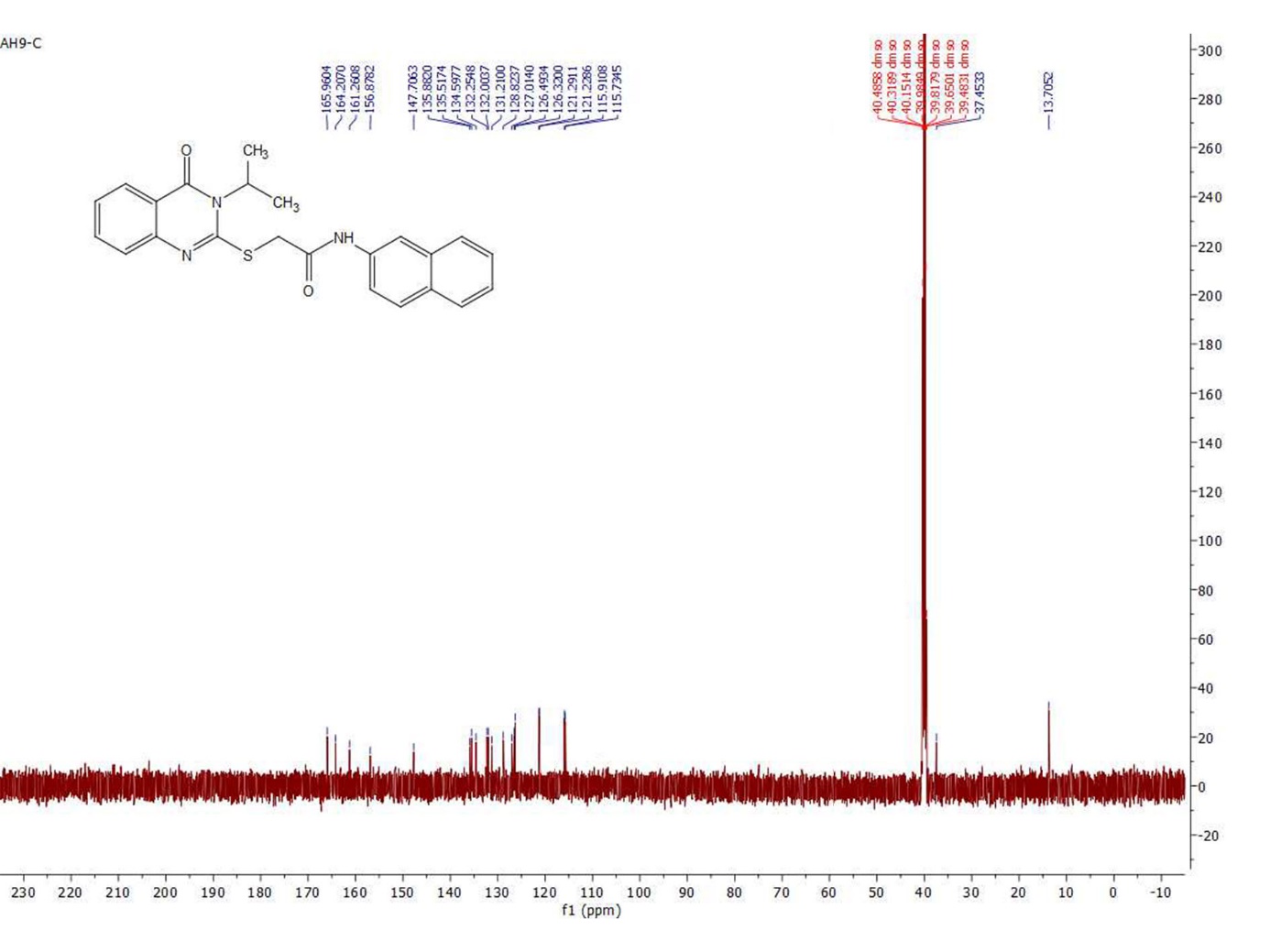


Figure S34. 1HNMR spectrum of compound 9p


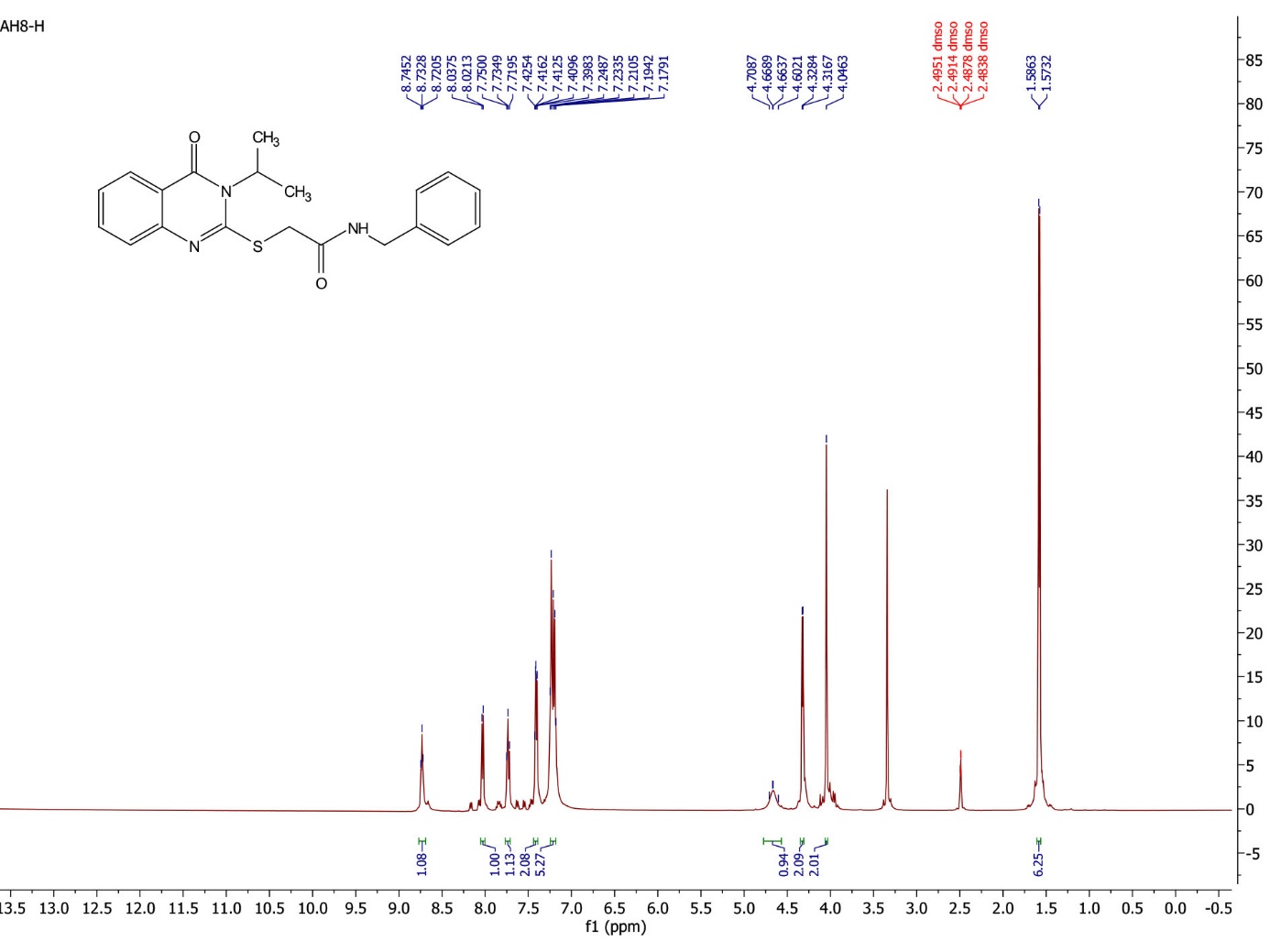


Figure S35. 13CNMR spectrum of compound 9p


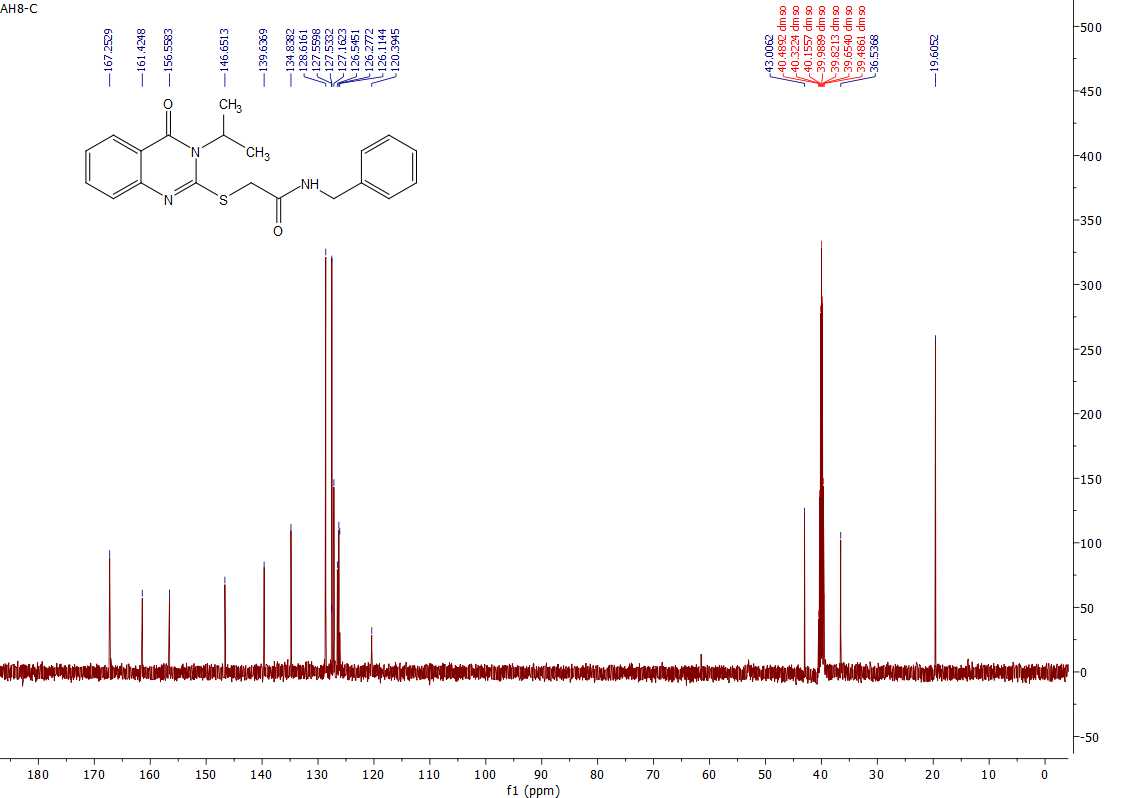


Figure S36. 1HNMR spectrum of compound 9q


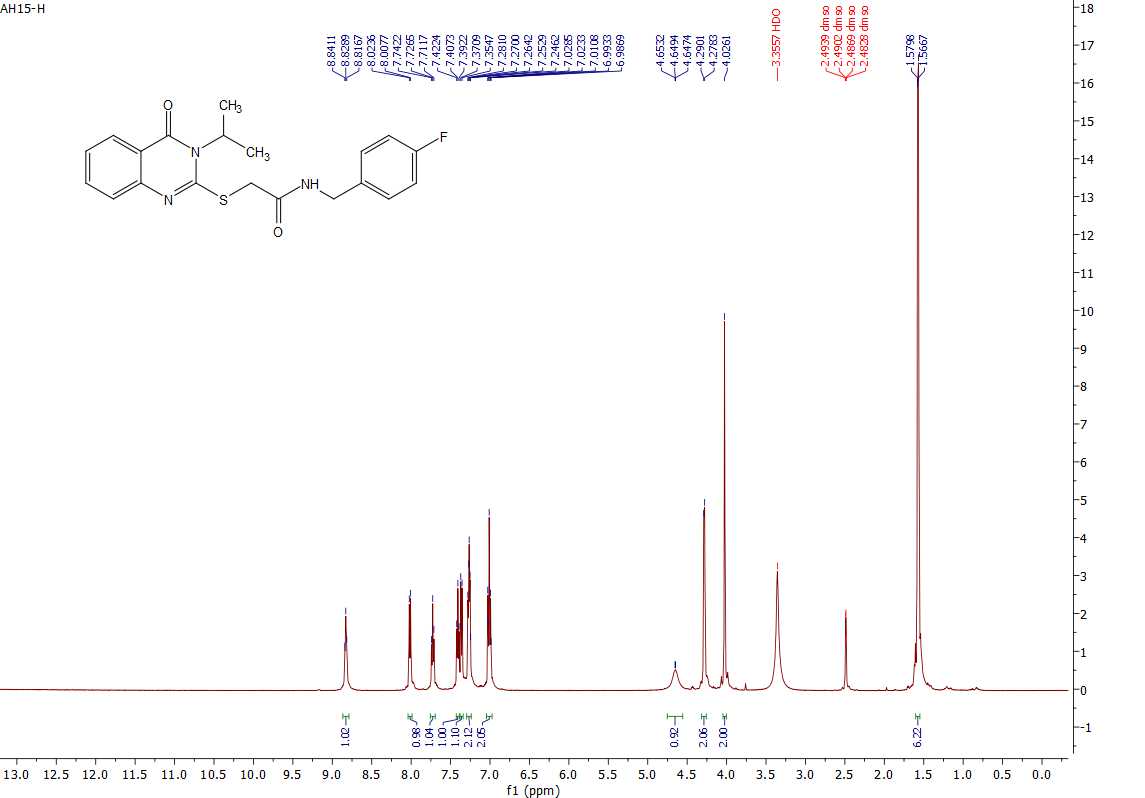


Figure S37. 13CNMR spectrum of compound 9q


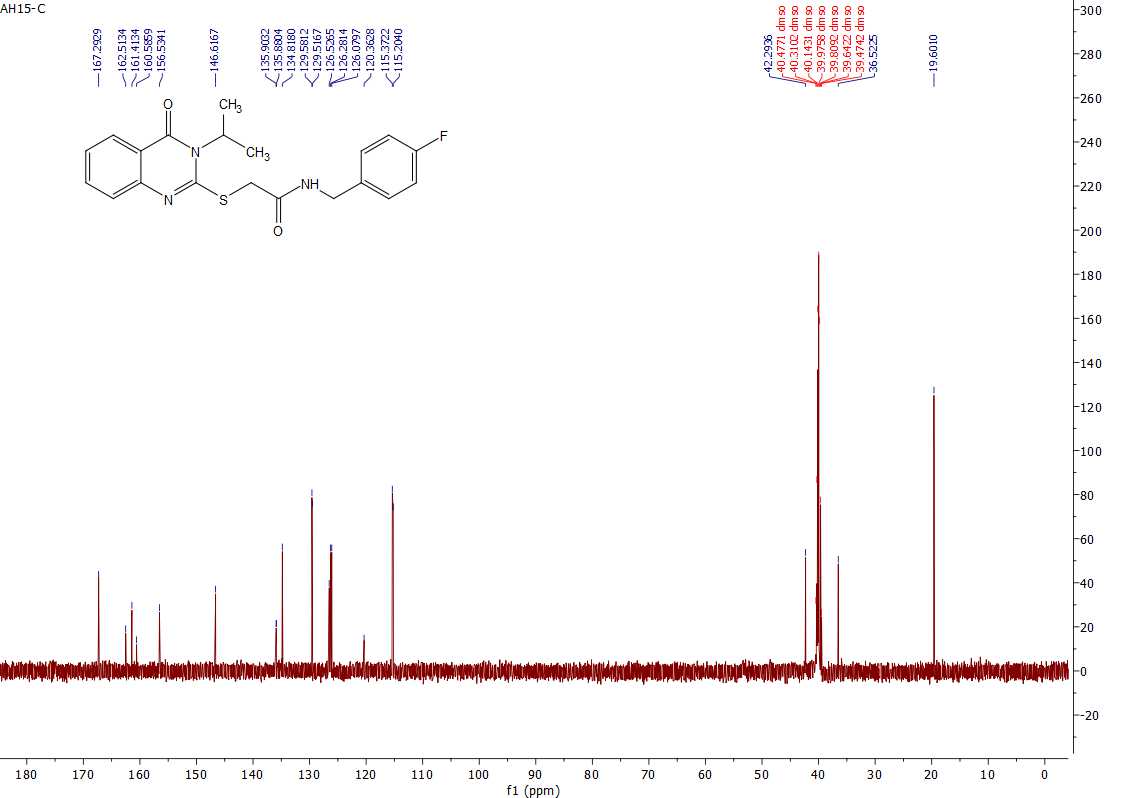

Supplement: Supplementary file 1 — Additional file 1: Figure. S1. HPLC results of 9a. Figure S2. HPLC results of 9n. Figure S3. HPLC results of 9p. Figure S4. 1HNMR spectrum of compound 9a. Figure S5. 13CNMR spectrum of compound 9a. Figure S6. 1HNMR spectrum of compound 9b. Figure S7. 13CNMR spectrum of compound 9b. Figure S8. 1HNMR spectrum of compound 9c. Figure S9. 13CNMR spectrum of compound 9c. Figure S10. 1HNMR spectrum of compound 9d. Figure S11. 13CNMR spectrum of compound 9d. Figure S12. 1HNMR spectrum of compound 9e. Figure S13. 13CNMR spectrum of compound 9e. Figure S14. 1HNMR spectrum of compound 9f. Figure S15. 13CNMR spectrum of compound 9f. Figure S16. 1HNMR spectrum of compound 9g. Figure S17. 13CNMR spectrum of compound 9g. Figure S18. 1HNMR spectrum of compound 9h. Figure S19. 13CNMR spectrum of compound 9h. Figure S20. 1HNMR spectrum of compound 9i. Figure S21. 13CNMR spectrum of compound 9i. Figure S22. 1HNMR spectrum of compound 9j. Figure S23. 13CNMR spectrum of compound 9j. Figure S24. 1HNMR spectrum of compound 9k. Figure S25. 13CNMR spectrum of compound 9k. Figure S26. 1HNMR spectrum of compound 9l. Figure S27. 13CNMR spectrum of compound 9l. Figure S28. 1HNMR spectrum of compound 9m. Figure S29. 13CNMR spectrum of compound 9m. Figure S30. 1HNMR spectrum of compound 9n. Figure S31. 13CNMR spectrum of compound 9n. Figure S32. 1HNMR spectrum of compound 9o. Figure S33. 13CNMR spectrum of compound 9o. Figure S34. 1HNMR spectrum of compound 9p. Figure S35. 13CNMR spectrum of compound 9p. Figure S36. 1HNMR spectrum of compound 9q. Figure S37. 13CNMR spectrum of compound 9q. [file 13065_2023_978_MOESM1_ESM.docx]
